# Integrated transcriptomic analysis of LMB2-induced podocyte injury identifies conserved inflammatory and adaptive stress responses

**DOI:** 10.1371/journal.pone.0352764

**Published:** 2026-07-31

**Authors:** Mohammad-Reza Esmaeili, Marjan Nejati, Shaqayeq Roqanian, Reza Moghadasali, Sara Taleahmad

**Affiliations:** 1 Department of Cell and Developmental Biology, Faculty of Basic Sciences and Advanced Technologies in Medicine, University of Science and Culture, Tehran, Iran; 2 Department of Stem Cells and Developmental Biology, Cell Science Research Center, Royan Institute for Stem Cell Biology and Technology, ACECR, Tehran, Iran; 3 Department of Cell and Molecular Biology, School of Biology, College of Sciences, University of Tehran, Tehran, Iran; 4 Department of Basic Science, Faculty of Veterinary Medicine, Science and Research Branch, Islamic Azad University, Tehran, Iran; Guangdong Nephrotic Drug Engineering Technology Research Center, Institute of Consun Co. for Chinese Medicine in Kidney Diseases, CHINA

## Abstract

Podocyte injury is a central driver of progressive glomerular diseases, yet the temporal organization of podocyte-intrinsic responses remains incompletely defined. In this study, we performed an integrated re-analysis of two publicly available mouse transcriptomic datasets (GSE108629 and GSE151869) to identify conserved molecular responses to LMB2-induced podocyte injury across comparable post-injury time points. Differential expression analysis was conducted independently at Day 4 and Day 7 in each dataset, followed by cross-dataset integration at each time point by identifying shared differentially expressed genes (DEGs) with consistent directionality. The resulting Day 4 and Day 7 shared gene sets were subsequently combined to define a final pooled shared DEG set. Functional enrichment, network analysis, upstream regulator prediction, and gene set enrichment analysis (GSEA) were used to characterize conserved biological processes and regulatory features. We identified 1,418 and 1,401 shared DEGs at Day 4 and Day 7, respectively, with 725 genes defining the final pooled shared DEGs set. At Day 4, the transcriptional response was characterized by inflammatory signaling and adaptive stress-related pathways, including endoplasmic reticulum stress. By Day 7, this response expanded to include extracellular matrix remodeling, focal adhesion reorganization, sustained inflammatory signaling, and downregulation of autophagy–lysosome pathways, together with disruption of ER-to-Golgi trafficking. Network analysis highlighted RELA-centered regulatory modules alongside epigenetic- and kinase-associated regulators. Collectively, these findings support a conserved, cross-dataset biphasic transcriptional response to podocyte injury, characterized by an early adaptive inflammatory phase followed by a later maladaptive state involving extracellular remodeling and impaired proteostasis. This framework provides a reproducible, temporally organized injury signature and is consistent with stage-specific pathways as potential targets for future mechanistic and therapeutic studies.

## Introduction

Podocyte injury is a major driver of progressive glomerular disease, including focal segmental glomerulosclerosis (FSGS) and nephrotic syndrome, and can ultimately lead to end-stage kidney failure. Podocytes are terminally differentiated epithelial cells with specialized foot processes that form the slit diaphragm and preserve the integrity of the glomerular filtration barrier. Because of their limited regenerative capacity, podocyte damage often results in irreversible loss of kidney function [1]. This process is a defining feature of primary glomerular diseases and is distinct from generalized acute kidney injury (AKI) [2]. Accordingly, LMB2-induced injury is generally regarded as a model of selective podocyte damage rather than a broad AKI model [3].

The endoplasmic reticulum (ER) is central to the maintenance of podocyte homeostasis. Podocytes contain multiple intracellular organelles, including lysosomes, extensive ER structures, and organized Golgi systems, all of which are required to sustain their complex architecture. Because the ER regulates the synthesis and folding of secretory proteins, insults such as oxidative stress or increased secretory demand can disrupt protein folding and induce ER stress [1,4–6]. Adaptation to ER stress is particularly important in podocytes because they are post-mitotic, terminally differentiated cells with limited renewal capacity. Persistent or unresolved ER stress may therefore impair podocyte function and structural integrity and contribute to a shift from adaptive to maladaptive cellular responses [1,4–6].

Experimental systems have been widely used to study podocyte injury, and LMB2 provides high selectivity through hCD25 targeting [7–10]. More recent LMB2-based mosaic and transgenic models have enabled more precise investigation of podocytopathy mechanisms [11–13]. Although AKI is a major clinical problem caused by diverse insults such as ischemia and toxins, LMB2-induced podocyte injury is generally considered a model of selective glomerular injury rather than a conventional AKI paradigm. Importantly, substantial podocyte loss can promote progression to chronic kidney disease (CKD) [14,15].

Despite the value of the LMB2 model, the temporal transition between early and late responses to podocyte injury remains incompletely defined [12]. In parallel, many patients with glomerular disease continue to progress to kidney failure and require dialysis or transplantation [16]. To address this gap, we integrated and re-analyzed two publicly available transcriptomic datasets, GSE108629 and GSE151869, generated from LMB2-treated podocytes. By comparing gene expression changes across comparable post-injury time points, we observed a conserved biphasic transcriptional pattern. The early phase (Day 4) was characterized by conserved inflammatory and adaptive stress-related responses, whereas the later phase (Day 7) showed sustained inflammatory signaling together with extracellular matrix remodeling, focal adhesion reorganization, and impaired autophagy-lysosome-associated processes. These findings provide insight into the temporal transcriptional landscape of podocyte injury and offer a framework for future hypothesis-driven studies.

## Materials and methods

### Data source and dataset selection

**Fig 1:**
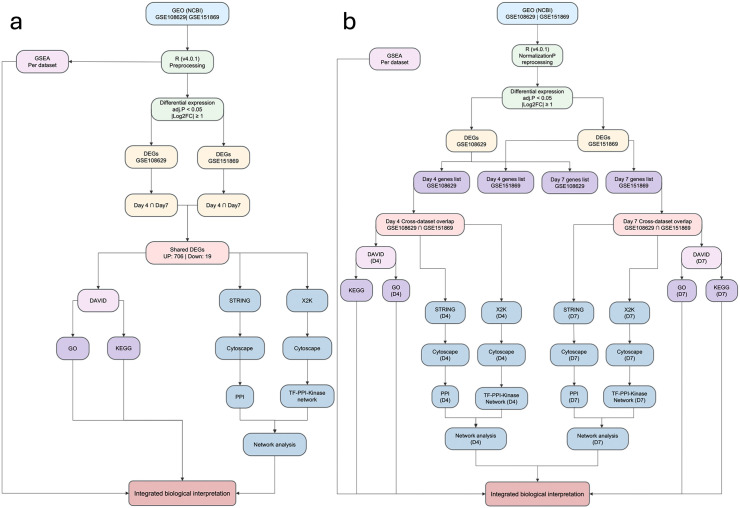
Integrated bioinformatic workflow of cross-dataset transcriptomic analysis in LMB2-induced podocyte injury. Public microarray datasets (GSE108629 and GSE151869) were obtained from GEO. After normalization and quality control, differential expression analyses were performed at Day 4 and Day 7. Shared DEGs were identified by intersection analysis and used for downstream GO, GSEA, X2K, and Cytoscape network analyses to identify conserved pathways and regulatory hubs.

Two mouse transcriptomic datasets, GSE108629 and GSE151869, were retrieved from the Gene Expression Omnibus (GEO) database. Both datasets were generated on the GPL10787 platform (Agilent-028005 SurePrint G3 Mouse GE 8 × 60K Microarray). Datasets were selected based on their relevance to podocyte injury, focal segmental glomerulosclerosis (FSGS), nephropathy, and the availability of corresponding control samples and comparable post-injury time points (Fig 1a, 1b).

GSE151869 comprised 29 samples collected at Day 0, Day 1, Day 4, and Day 7 following LMB2-induced podocyte injury. Day 0 samples served as the pre-injury control group, and TdTomato-positive podocytes were defined as injured podocytes after LMB2 administration. For cross-dataset analyses, only the comparable post-injury time points, Day 4 and Day 7, were retained because GSE108629 did not include a corresponding Day 1 time point. In GSE151869, Day 0 samples were used as the common reference control for both the Day 4 and Day 7 comparisons. At the selected time points, injured podocytes were analyzed at n = 4 per group, except for Day 7 TdTomato-positive samples (n = 2).

GSE108629 included 16 samples comprising normal podocytes as the control group (n = 4) and LMB2-treated podocytes collected at Day 4 and Day 7 (n = 4 per time point). In this dataset, normal podocytes served as the reference control group for both the Day 4 and Day 7 comparisons.

### Data preprocessing and differential expression analysis

All analyses were performed in R (version 4.0.1). Processed expression data were retrieved from GEO using GEOquery and quantile-normalized using limma. Probe identifiers were mapped to gene symbols using the GPL10787 platform annotation downloaded from the corresponding GEO platform record (Agilent-028005 SurePrint G3 Mouse GE 8 × 60K Microarray). When multiple probes mapped to the same gene symbol, the probe with the highest mean expression was retained.

Data quality was assessed by boxplot inspection and principal component analysis (PCA) before and after normalization. Differential expression patterns were visualized using volcano plots and heatmaps (S1 Fig, S2 Fig).

Differential expression analysis was performed using linear models implemented in limma. For GSE151869, injured podocytes at Day 4 and Day 7 were each compared with the shared Day 0 control group. For GSE108629, LMB2-treated podocytes at Day 4 and Day 7 were each compared with the shared normal podocyte control group. Statistical significance was assessed using empirical Bayes moderation. Genes with an adjusted P-value < 0.05 and |log2 fold change| ≥ 1 were considered differentially expressed.

Each dataset was analyzed independently. Batch correction across the two datasets was not applied because the analyses were designed at the within-dataset level rather than on a merged expression matrix. To minimize cross-study technical confounding while preserving biologically concordant signals, cross-dataset integration was performed only after differential expression analysis by retaining shared genes with consistent regulation at comparable post-injury time points.

### Identification of shared differentially expressed genes

To identify conserved injury-associated transcriptional responses, cross-dataset overlap analysis was performed separately at Day 4 and Day 7. Differentially expressed genes (DEGs) were identified independently in GSE108629 and GSE151869 at each time point relative to the corresponding control group. Upregulated and downregulated genes were analyzed separately.

For each time point, DEG lists from the two datasets were intersected, and only genes with a consistent direction of regulation were retained. This procedure generated shared upregulated and shared downregulated gene sets for Day 4 and Day 7.

### Construction of the final pooled shared DEG set

Shared upregulated genes identified at Day 4 and Day 7 were combined into one set, and shared downregulated genes from the same time points were combined into another set. These two sets were then merged to generate the final pooled shared DEG set.

Time point-specific shared DEG sets and the final pooled shared DEG set were used for downstream analyses to prioritize transcriptional changes that were reproducibly detected across both datasets. This approach reduced the influence of dataset-specific signals and enabled the identification of conserved molecular responses associated with LMB2-induced podocyte injury across comparable post-injury time points.

The final pooled shared DEG set was used for downstream functional enrichment and network analyses. Functional enrichment and protein-protein interaction analyses were performed using DAVID and STRING, respectively. In addition, eXpression2Kinases (X2K) analysis was performed on the Day 4 shared DEG set, the Day 7 shared DEG set, and the final pooled shared DEG set. Results from these analyses are presented in the Supplementary Materials.

### Functional enrichment analysis

Functional enrichment analysis of the final pooled shared DEG set was performed using DAVID (version 6.8). Gene Ontology enrichment analysis was conducted separately for upregulated and downregulated genes across the categories of biological process, cellular component, and molecular function. Kyoto Encyclopedia of Genes and Genomes pathway analysis was also performed. Enrichment results with P-value < 0.05 were considered significant.

### Protein–protein interaction network analysis

Protein–protein interaction networks were constructed from the final pooled shared DEG set using STRING (version 11.5) for Mus musculus. Interactions were filtered at a high-confidence score threshold (≥ 0.9). The default STRING functional association network was used with all available evidence sources enabled. Isolated nodes were excluded before export, and STRING confidence scores were retained as edge weights.

The exported interaction networks were visualized in Cytoscape (version 3.9.1), with log2 fold change values mapped to nodes. Network topology was evaluated using cytoHubba and CentiScaPe (version 2.2). Hub gene prioritization was performed by first ranking nodes according to degree and then refining the ranking using betweenness centrality as a secondary criterion. The top 10 ranked genes were designated as hub genes.

### Upstream regulator analysis

Upstream regulator analysis was performed using X2K on three shared DEG sets: the Day 4 shared DEG set, the Day 7 shared DEG set, and the final pooled shared DEG set. The Day 4 and Day 7 sets each comprised the common differentially expressed genes identified in both GSE108629 and GSE151869 at the corresponding time point, regardless of direction of regulation. The final pooled shared DEG set was generated by merging the shared DEG sets from Day 4 and Day 7. For all three analyses, the default X2K workflow was applied without additional parameter modification, including transcription factor enrichment, protein–protein interaction network expansion, and kinase enrichment. The resulting regulatory networks were visualized in Cytoscape, and the identified factors were interpreted as predicted upstream regulatory candidates. Full results are provided in the Supplementary Materials.

### Gene set enrichment analysis (GSEA)

Gene set enrichment analysis (GSEA) was performed independently for each dataset at each comparable time point using GSEA software (version 4.3.2) on quantile-normalized expression matrices without prior gene filtering. Phenotype labels were defined according to the corresponding group comparisons (control vs Day 4 and control vs Day 7). Gene sets were obtained from the Reactome collection. Genes were ranked using the Signal2Noise metric, and enrichment analysis was performed on the full ranked gene list. Gene identifiers were collapsed to gene symbols using the GPL10787 chip file with the “collapse dataset to gene symbols” option enabled. Pathways with a false discovery rate q-value < 0.25 were considered significantly enriched. Enrichment results were interpreted at the pathway level.

## Results

### Cross-dataset transcriptomic separation and identification of shared differentially expressed genes in LMB2-injured podocytes

Principal component analysis (PCA) and hierarchical clustering heatmaps showed clear separation of LMB2-injured and control podocyte samples in both GSE108629 and GSE151869, indicating a consistent injury-associated transcriptomic shift across the two datasets (S1 Fig a-c). Day 4 and Day 7 were the only comparable post-injury time points shared between the two datasets and were therefore selected for cross-dataset differential expression analysis.

**Fig 2:**
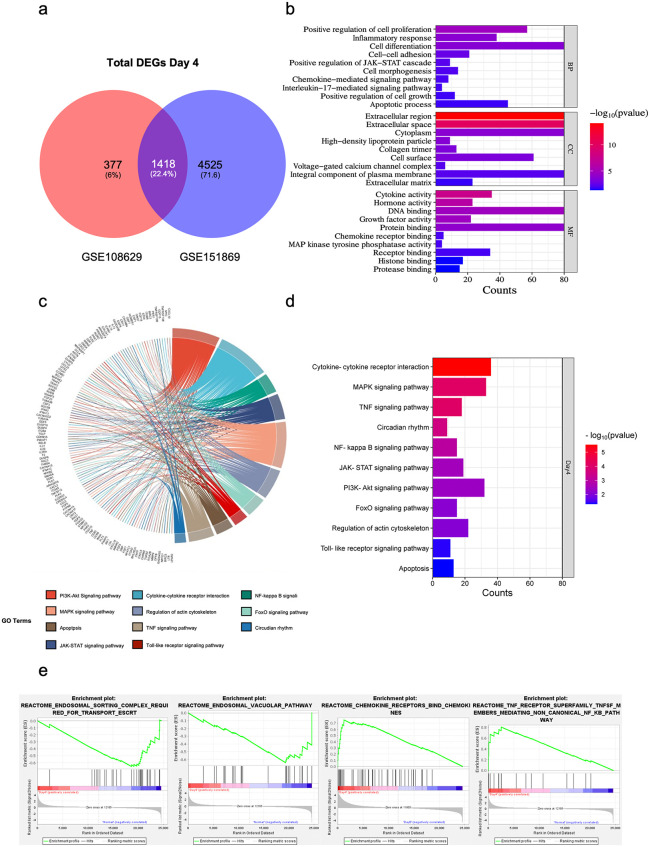
Functional enrichment analysis of shared differentially expressed genes (DEGs) at Day 4. (A) Venn diagram showing overlapping DEGs between GSE108629 and GSE151869 at Day 4. (B) Gene Ontology (GO) enrichment analysis of biological process (BP), cellular component (CC), and molecular function (MF). (C) Chord plot showing associations between DEGs and enriched KEGG pathways. (D) Top enriched KEGG pathways identified at Day 4. (E) Gene set enrichment analysis (GSEA) plots showing significantly enriched pathways.

**Fig 3:**
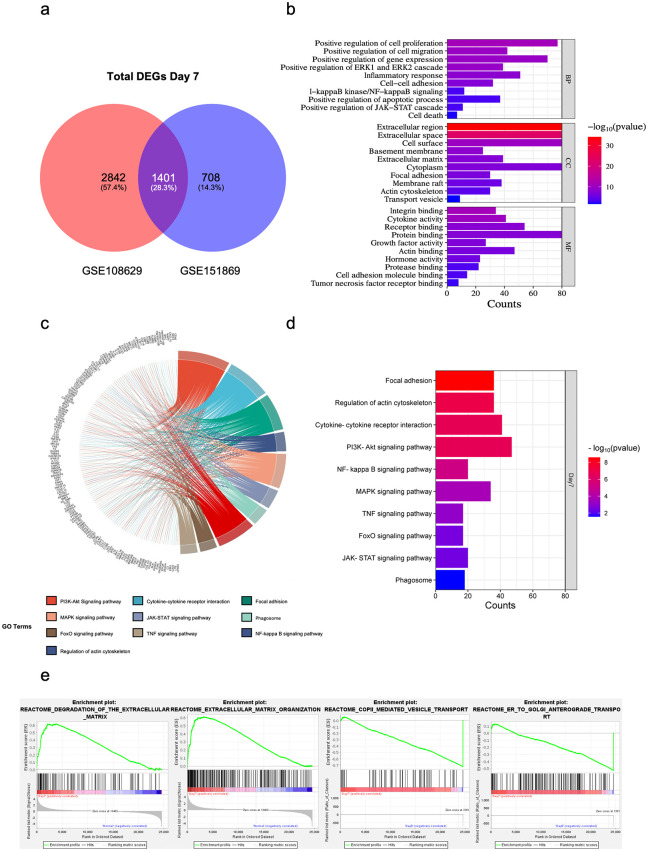
Functional enrichment analysis of shared differentially expressed genes (DEGs) at Day 7. (A) Venn diagram showing overlapping DEGs between GSE108629 and GSE151869 at Day 7. (B) Gene Ontology (GO) enrichment analysis of biological process (BP), cellular component (CC), and molecular function (MF). (C) Chord plot showing associations between DEGs and enriched KEGG pathways. (D) Top enriched KEGG pathways identified at Day 7. (E) Gene set enrichment analysis (GSEA) plots showing significantly enriched pathways.

Cross-dataset comparison identified 1,418 shared DEGs at Day 4 (Fig 2a) and 1,401 shared DEGs at Day 7 (Fig 3a). The overlap between these two shared DEG sets comprised 725 genes and was defined as the final pooled shared DEG set. The final pooled shared DEG set comprised 706 upregulated and 19 downregulated genes (Fig 3a, 3b). Volcano plots for each dataset at Day 4 and Day 7 illustrated the magnitude and significance of differential expression, with the top ten most significant DEGs labeled in each panel’s plot (S2 Fig). Detailed numbers of upregulated and downregulated genes identified in each dataset and time point are available in (S1 Table).

### A conserved early transcriptional injury response is detected across datasets at Day 4

At Day 4, the shared DEG set revealed a conserved early transcriptional response to LMB2-induced podocyte injury across datasets. Functional enrichment analysis showed enrichment of inflammatory response, chemokine-mediated signaling pathway, interleukin-17-mediated signaling pathway, and positive regulation of the JAK-STAT cascade (Fig 2c,2d). Cell differentiation and positive regulation of cell proliferation were also among the enriched biological processes (BP) (Fig 2b), consistent with activation of an early injury-responsive transcriptional program. In addition, upregulation of *ATF3*, *ATF4*, *ATF5*, *PDIA4*, and multiple heat-shock protein (HSP) family members was consistent with activation of adaptive stress-related pathways and ER stress-related responses.

Among upregulated genes, cellular component (CC) and molecular function (MF) analyses revealed that 43.83% of upregulated genes localized to the nucleus and nucleoplasm and were associated with DNA binding and activator/regulator functions linked to RNA polymerase II (RNAP II)-mediated transcription, whereas 33.82% were cytoplasmic and 18.10% were localized to the cell surface and receptor complexes. These findings support coordinated transcriptional and signal transduction programs across multiple cellular compartments (Fig 2b, S3 Fig a-e, S4 Fig a). By contrast, downregulated DEGs at Day 4 were primarily associated with cell adhesion, apoptotic processes, and endocytosis pathways (Fig 2b,2c, S3 Fig b, e, S4 Fig b).

GSEA further supported the Day 4 inflammatory program, highlighting chemokine receptor binding, regulation of IFN-α/β signaling, TNF superfamily members mediating non-canonical NF-κB activation, and ligand uptake by scavenger receptors (Fig 2e). Endosomal trafficking pathways were concurrently suppressed, consistent with early disruption of vesicular homeostasis in the context of marked inflammatory activation at this stage. Together, these findings indicate that at Day 4, podocyte injury was characterized by a conserved early transcriptional program associated with inflammatory activation and adaptive stress responses.

### A sustained and expanded conserved injury program is detected across datasets at Day 7

At Day 7, the shared DEG set revealed a sustained and expanded conserved injury response across datasets, characterized by enrichment of structural remodeling, extracellular matrix organization, and focal adhesion pathways (Fig 3b-3d, S3 Fig f, S4 Fig c, d). Consistent with the Day 4 profile, inflammatory signaling remained evident at this stage, as reflected by enrichment of inflammatory response, I-kappaB kinase/NF-kappaB signaling, and positive regulation of the JAK-STAT and ERK1/2 cascades, together with cell death- and apoptosis-related processes (Fig 3b-3d). CC and MF analyses further showed enrichment of extracellular matrix, basement membrane, focal adhesion, actin cytoskeleton, membrane raft, cell surface, cytokine activity, and TNFR binding, consistent with extracellular remodeling and adhesion-related reorganization at Day 7 (Fig 3b, S3 Fig e, S4 Fig c, d).

GSEA further indicated downregulation of lysosomal and autophagy-related pathways, together with negative enrichment of ESCRT complexes, the ER-phagosome pathway, COPII-mediated vesicle budding, and ER-to-Golgi anterograde transport, whereas retrograde ER-to-Golgi transport was enriched (Fig 3e). These changes were consistent with unresolved chronic ER stress and persistent disruption of proteostasis, stress-associated intracellular trafficking, and the cellular protein quality control machinery at Day 7. Notably, the marked downregulation of core components such as *Chmp4b*, a component of the ESCRT-III complex, further supported a sustained blockade of autophagy-lysosome flux. Together, these findings indicate that the conserved Day 7 injury program combined sustained inflammatory signaling with extracellular matrix remodeling, focal adhesion reorganization, impaired autophagy-lysosome function, and a broader shift from acute inflammation toward a late maladaptive state in injured podocytes.

### Integration of Day 4 and Day 7 shared signatures defines the final pooled shared DEG set as a core LMB2-induced podocyte injury signature

**Fig 4:**
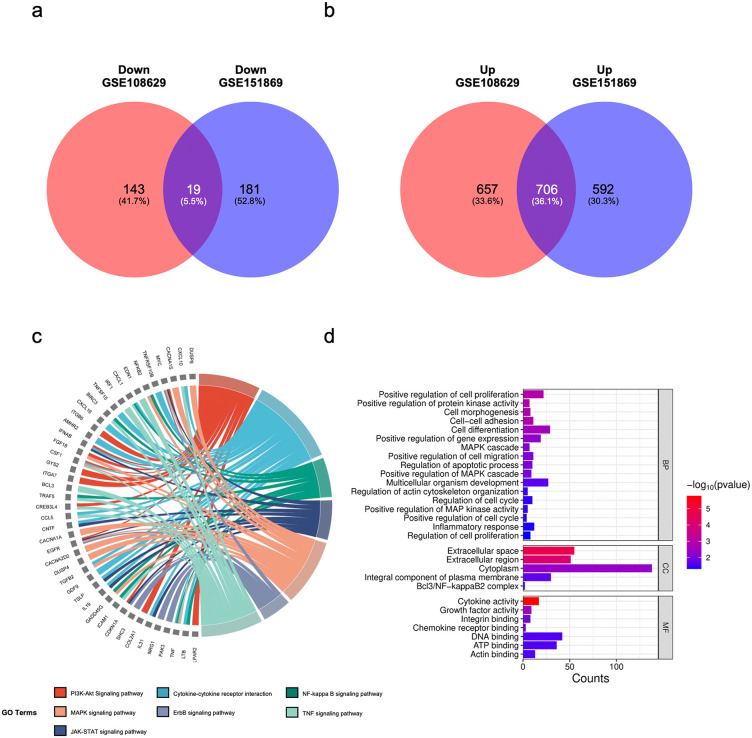
Functional enrichment analysis of the final pooled shared differentially expressed gene (DEG) set. (A) Venn diagram showing the overlap between Day 4 and Day 7 shared DEG sets. (B) Distribution of upregulated and downregulated genes in the final pooled shared DEG set. (C) Chord plot showing associations between pooled DEGs and enriched KEGG pathways. (D) Gene Ontology (GO) enrichment analysis of biological process (BP), cellular component (CC), and molecular function (MF.

The overlap between the two shared DEG sets identified at Day 4 and Day 7 comprised 725 genes and was defined as the final pooled shared DEG set. This pooled signature comprised 706 upregulated genes and 19 downregulated genes (Fig 4a, 4b). Functional enrichment analysis showed enrichment of BPs related to inflammatory response, regulation of cell differentiation, proliferation, migration, cell-cell adhesion, regulation of apoptotic process, and MAPK-associated signaling (Fig 4d). CC analysis further indicated enrichment of the cytoplasm, extracellular region, extracellular space, integral component of the plasma membrane, and the Bcl3/NF-kappaB2 complex, whereas MF analysis highlighted DNA binding, cytokine and growth factor activity, and integrin/actin binding (Fig 4d). Together, these findings link a cytokine/inflammatory signaling axis to proliferative and apoptotic regulation, as well as cytoskeletal and extracellular matrix remodeling.

### Network topology and upstream regulator analyses reveal distinct Day 4, Day 7, and pooled regulatory features of the conserved injury signature

**Fig 5:**
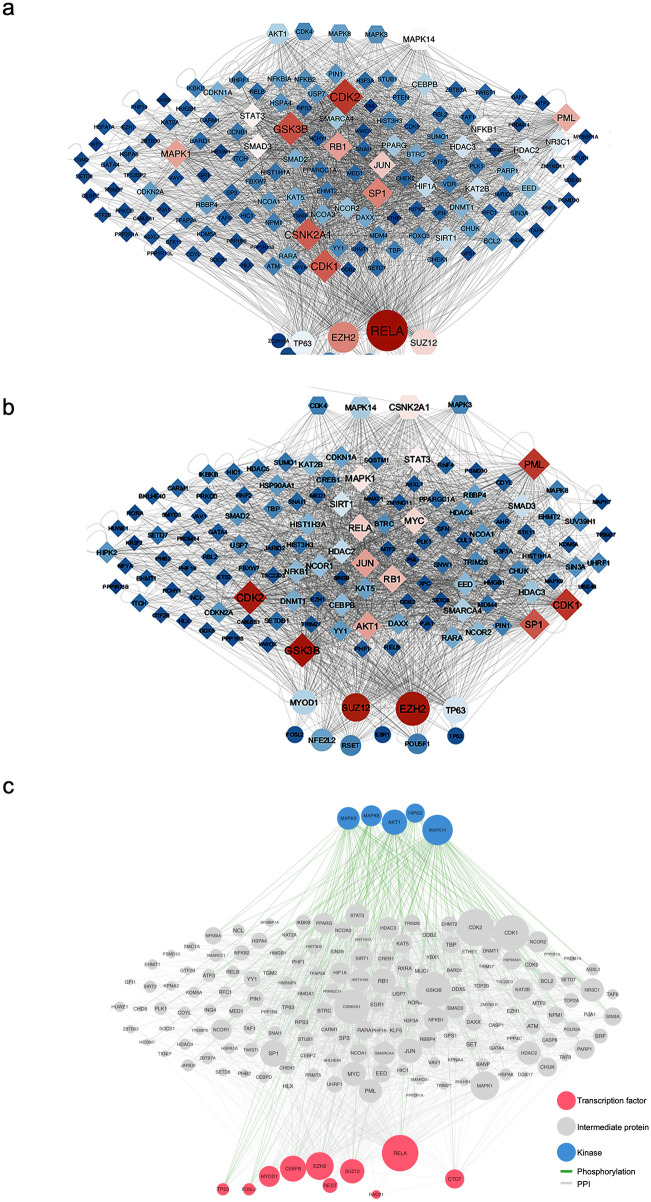
Network topology and upstream regulator analysis of shared podocyte injury signatures. Protein–protein interaction–transcription factor–protein kinase (PPI-TF-PK) networks were generated using eXpression2Kinases (X2K). (A) Day 4 shared DEG regulatory network. (B) Day 7 shared DEG regulatory network. (C) Final pooled shared DEG regulatory network.

Predicted upstream regulator networks were generated using eXpression2Kinases (X2K) for the shared DEG sets from Day 4, Day 7, and the final pooled shared DEG set. In the Day 4 PPI-TF-PK network, RELA was the most prominent transcription factor node, together with *EZH2* and *SUZ12*, while the kinase layer included *MAPK14*, *AKT1*, *CDK2*, and *GSK3B* (Fig 5a). In the Day 7 network, *RELA*, *EZH2*, *SUZ12*, and *MYOD1* were prominent transcription factor nodes, with continued representation of *MAPK14*, *AKT1*, *CDK1*, *CDK2*, and *GSK3B* in the associated regulatory layer (Fig 5b).

In the pooled analysis, the PPI-TF network highlighted *RELA*, *EZH2*, *CEBPB*, *SUZ12*, *MYOD1*, *FOXM1*, and *REST* as prominent transcription factor nodes. Incorporation of the kinase layer in the pooled PPI-TF-PK network further identified *MAPK14*, *AKT1*, *MAPK3*, *MAPK8*, and *HIPK2* as major kinase-associated regulators (Fig 5c).

Together, these predicted networks suggest RELA-centered inflammatory regulation, together with epigenetic and kinase-linked regulatory inputs across the conserved injury signature. Topologically prioritized hub genes identified by network analysis using Cytoscape are presented in S1 Table.

## Discussion

LMB2-induced podocyte injury in the NEP25 mouse model is associated with a reproducible biphasic transcriptional pattern, as revealed by integrated re-analysis of two independent datasets (GSE108629 and GSE151869) [11,13]. Cross-dataset comparison identified 1,418 and 1,401 shared DEGs at Day 4 and Day 7, respectively, with 725 overlapping genes defining a final pooled shared DEG set enriched for conserved injury-associated responses. By prioritizing shared transcriptional changes, this approach captures core molecular programs underlying podocyte injury. Given the limited proliferative capacity of podocytes, their loss is a key determinant of glomerular barrier dysfunction in diseases such as focal segmental glomerulosclerosis (FSGS), membranous nephropathy (MN), and diabetic nephropathy (DN) [1]. The relative selectivity of the LMB2 model therefore provides a suitable framework for examining podocyte-specific injury responses [2,3,17–20].

Across both day-specific and pooled DEG analyses, inflammatory signaling pathways emerged as dominant features of the transcriptional response, consistent with the early phase of the proposed biphasic injury model in which injury-triggered inflammatory activation represents the initial response. Persistent enrichment of TNF/NF-κB-associated signatures within the pooled shared DEG set indicates a conserved inflammatory core reproducible across datasets and time points. Consistent representation of Tnf and Ltb supports their involvement in this response, in line with established roles in cytokine signaling and NF-κB activation in renal inflammation [21–23]. Enrichment of chemokine signaling [24], interleukin-17-related pathways [25], *Csf1*, *Stat3* and *IFN-α/β* and *TNF*-associated transcriptional programs at Day 4 further supports inflammatory activation as an early and stable component of the injury response [26–29]. These findings suggest that inflammatory activation may contribute to downstream maladaptive responses [3], potentially through NF-κB-mediated transcriptional amplification of cytokine signaling [28], suggesting a potential link between inflammatory signaling and downstream proteostatic and structural alterations [30]. While these patterns are consistent with upstream innate immune involvement, specific pathways such as Toll-like receptor signaling cannot be directly inferred from transcriptomic data alone.

In parallel, Day 4 was characterized by activation of adaptive stress-related pathways, further supporting the early phase of the biphasic model defined by concurrent inflammatory activation and stress adaptation. Upregulation of *ATF3*, *ATF4*, *ATF5*, and multiple heat-shock proteins indicates engagement of ER stress–associated responses and early proteostatic adaptation [31–34], where increased expression of ATF family transcription factors may promote transcriptional programs that enhance protein folding capacity and stress-responsive gene expression to temporarily maintain cellular homeostasis under injury conditions, consistent with stress adaptation in terminally differentiated cells such as podocytes [28,31].

Network analysis identified RELA [35,36] as a central transcriptional regulator across Day 4, Day 7, and pooled DEG sets, alongside epigenetic-associated factors (*EZH2*, *SUZ12*) [29,37] and kinase regulators (*MAPK14*, *AKT1*, *GSK3B*) [38–40], providing a regulatory framework that may operate across both phases of the biphasic injury response [35]. This supports sustained NF-κB–centered transcriptional control integrated with kinase signaling and chromatin-associated regulatory mechanisms [29]. While epigenetic contributions cannot be directly inferred, their consistent association suggests coordinated multi-layer regulation of the injury response, similar to regulatory architectures reported in inflammatory injury contexts [37]; further investigation is required to determine how these epigenetic regulators functionally contribute to transcriptional reprogramming in injured podocytes [28].

At Day 7, the transcriptional landscape shifted toward extracellular matrix organization, focal adhesion, and cytoskeletal pathways, marking the transition into the late phase of the biphasic model, characterized by sustained stress, structural remodeling, and progressive maladaptation [41,42]. This shift was accompanied by continued inflammatory signaling and increased representation of apoptosis-related processes, indicating that persistent stress and inflammatory cues converge to promote cellular dysfunction [36,39]. Mechanistically, such changes may disrupt slit diaphragm integrity and cytoskeletal stability, contributing to progressive loss of filtration barrier function [43].

The Day 7 negative enrichment of ESCRT- and COPII-associated pathways suggests attenuation of vesicular trafficking and membrane-homeostatic transcriptional programs during late LMB2-induced podocyte injury. COPII-mediated trafficking supports ER export of newly synthesized membrane and secretory cargoes toward ERGIC/Golgi compartments, and recent work emphasizes that COPII coat assembly and cargo recognition are adaptable processes required to accommodate diverse secretory demands [44,45]. In parallel, ESCRT complexes mediate post-endocytic cargo sorting, intraluminal vesicle/multivesicular body formation, and lysosome-directed degradation of membrane-associated cargo [46,47]. Reduced representation of these pathways may therefore reflect diminished transcriptional support for both secretory delivery/replacement and endosomal processing of membrane-associated proteins.

This interpretation is particularly relevant to podocytes because filtration-barrier integrity depends on regulated membrane-protein delivery, surface localization, retrieval, recycling, degradation, slit diaphragm organization, and foot process architecture [48,49]. Slit-diaphragm proteins such as nephrin and podocin require regulated localization and turnover; CIN85/RukL mediates nephrin/podocin-associated slit diaphragm turnover, and recent podocyte/nephrocyte work shows that disruption of endocytosis impairs slit diaphragm integrity, increases oxidative stress, and disrupts nephrin/podocin-associated protein interactions [50,51].

Thus, attenuation of ESCRT/COPII-associated programs may indicate reduced membrane-homeostatic capacity in injured podocytes, potentially increasing vulnerability to reduced membrane turnover, impaired cargo clearance, proteostatic stress, and structural destabilization, particularly in the context of concurrent lysosomal and autophagy-related pathway suppression [52–54]. However, these findings remain pathway-level transcriptomic evidence and should not be interpreted as direct proof of ESCRT/COPII complex inactivation, impaired vesicular flux, or defective nephrin/podocin trafficking.

Conceptually, these results support a temporally coordinated biphasic model of podocyte injury in which early inflammatory activation and adaptive stress responses (Day 4) transition into a late maladaptive state characterized by sustained inflammation, extracellular remodeling, proteostasis disruption, and apoptotic signaling (Day 7) [52]. This temporal organization provides a coherent framework linking early adaptive responses to progressive cellular dysfunction under sustained injury conditions [43].

Taken together, the pooled shared DEG set defines a conserved transcriptional program integrating cytokine-driven inflammation with stress adaptation, proliferative and apoptotic regulation, and cytoskeletal and extracellular remodeling. RELA-centered regulatory networks, together with kinase and epigenetic-associated nodes, highlight coordinated control of injury responses and identify candidate regulators for future mechanistic studies [29,35]. These findings suggest that stage-specific targeting of inflammatory versus stress-adaptive pathways may represent a potential strategy for preserving podocyte function [38].

These findings should be interpreted within the limitations of a retrospective bulk transcriptomic analysis. The microarray-based design does not permit direct inference of causality, protein activity, or cell-type–specific mechanisms, and contributions from non-podocyte populations cannot be excluded. Accordingly, these results are hypothesis-generating and require experimental validation in selective podocyte injury models.

AI use

The authors used AI-assisted language editing to improve readability, basic grammar, and spelling check; all scientific content, analysis, and conclusions were generated and verified by the authors.

## Supporting information

S1 Fig Quality control and exploratory data analysis of transcriptomic datasets. (A) Boxplots of normalized expression data. (B) Hierarchical clustering heatmap. (C) Principal Component Analysis (PCA) plot.
(TIFF)

S2 Fig Volcano plots of DEGs across datasets. (A) Volcano plot for GSE108629. (B) Volcano plot for GSE151869. Red/green dots indicate significantly up- and downregulated genes, respectively.
(TIFF)

S3 Fig Comparative enrichment analysis at Day 4 and Day 7. (A–D) Venn diagrams of up- and downregulated DEGs at Day 4 (A–B) and Day 7 (C–D). (E–F) Top enriched GO terms and KEGG pathways.
(TIFF)

S4 Fig Cellular Component (CC) and Molecular Function (MF) GO enrichment. (A–B) CC and MF enrichment of shared DEGs at Day 4. (C–D) CC and MF enrichment of shared DEGs at Day 7.
(TIFF)

S5 Fig PPI networks by dataset and time point. (A–B) PPI networks at Day 4. (C–D) PPI networks at Day 7. Node size and color represent degree and fold-change, respectively.
(TIFF)

S1 Table Integration of Day 4 and Day 7 shared signatures. (XLSX)
