# Supplementary figures and images for: Integrated transcriptomic analysis of LMB2-induced podocyte injury identifies conserved inflammatory and adaptive stress responses
Source: PLoS One. 2026 Jul 31;21(7):e0352764. doi: 10.1371/journal.pone.0352764 (PMC13426963; doi:10.1371/journal.pone.0352764)

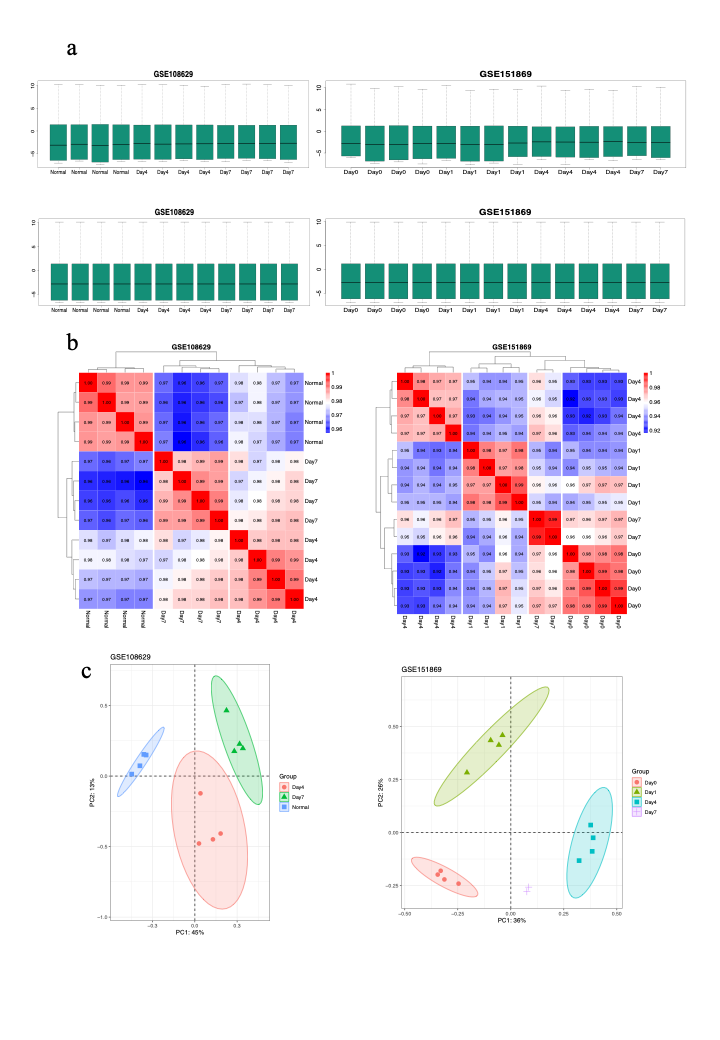

Supplement: S1 Fig — (A) Boxplots of normalized expression data. (B) Hierarchical clustering heatmap. (C) Principal Component Analysis (PCA) plot. (TIFF) [file pone.0352764.s001.tiff]

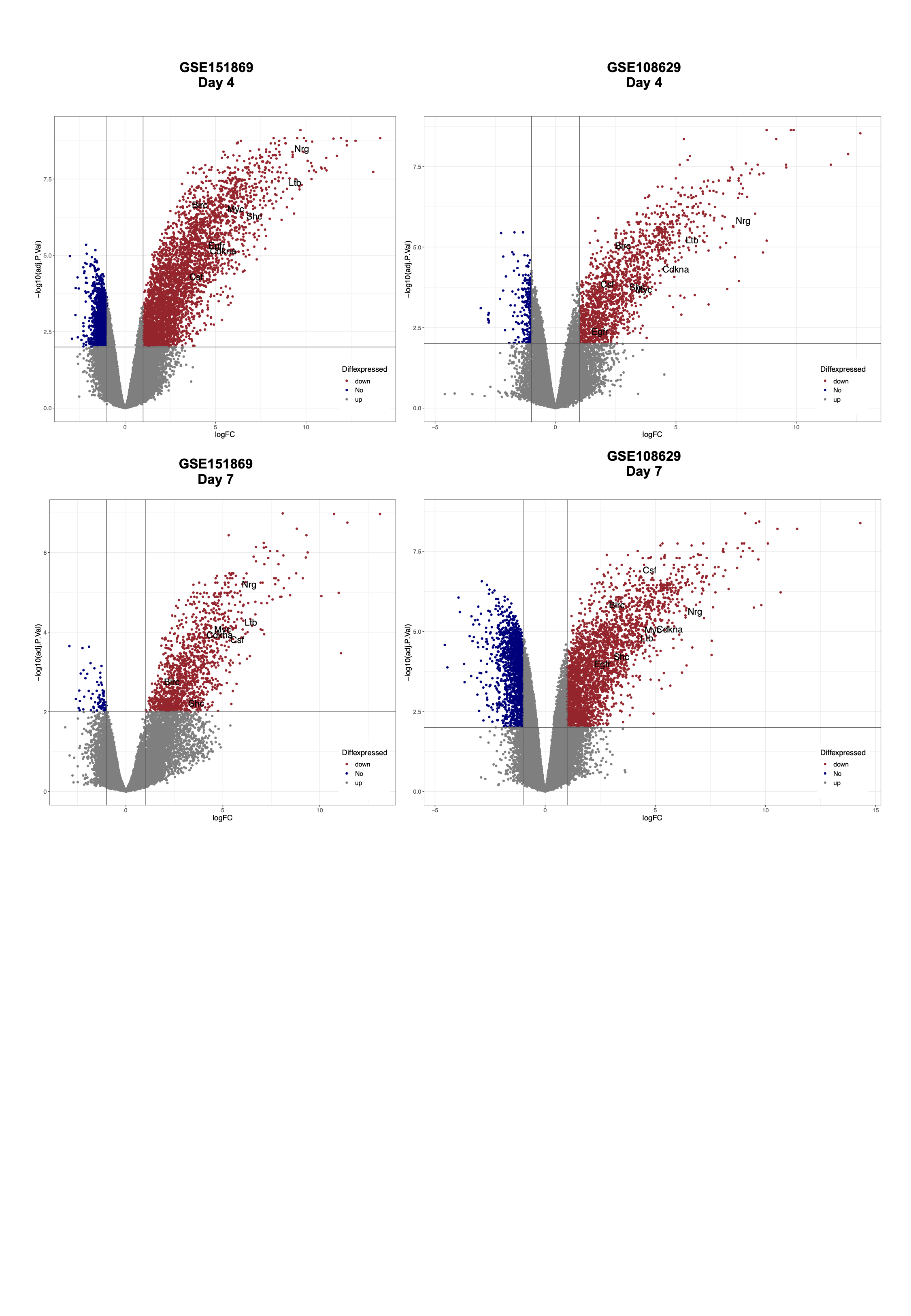

Supplement: S2 Fig — (A) Volcano plot for GSE108629. (B) Volcano plot for GSE151869. Red/green dots indicate significantly up- and downregulated genes, respectively. (TIFF) [file pone.0352764.s002.tiff]

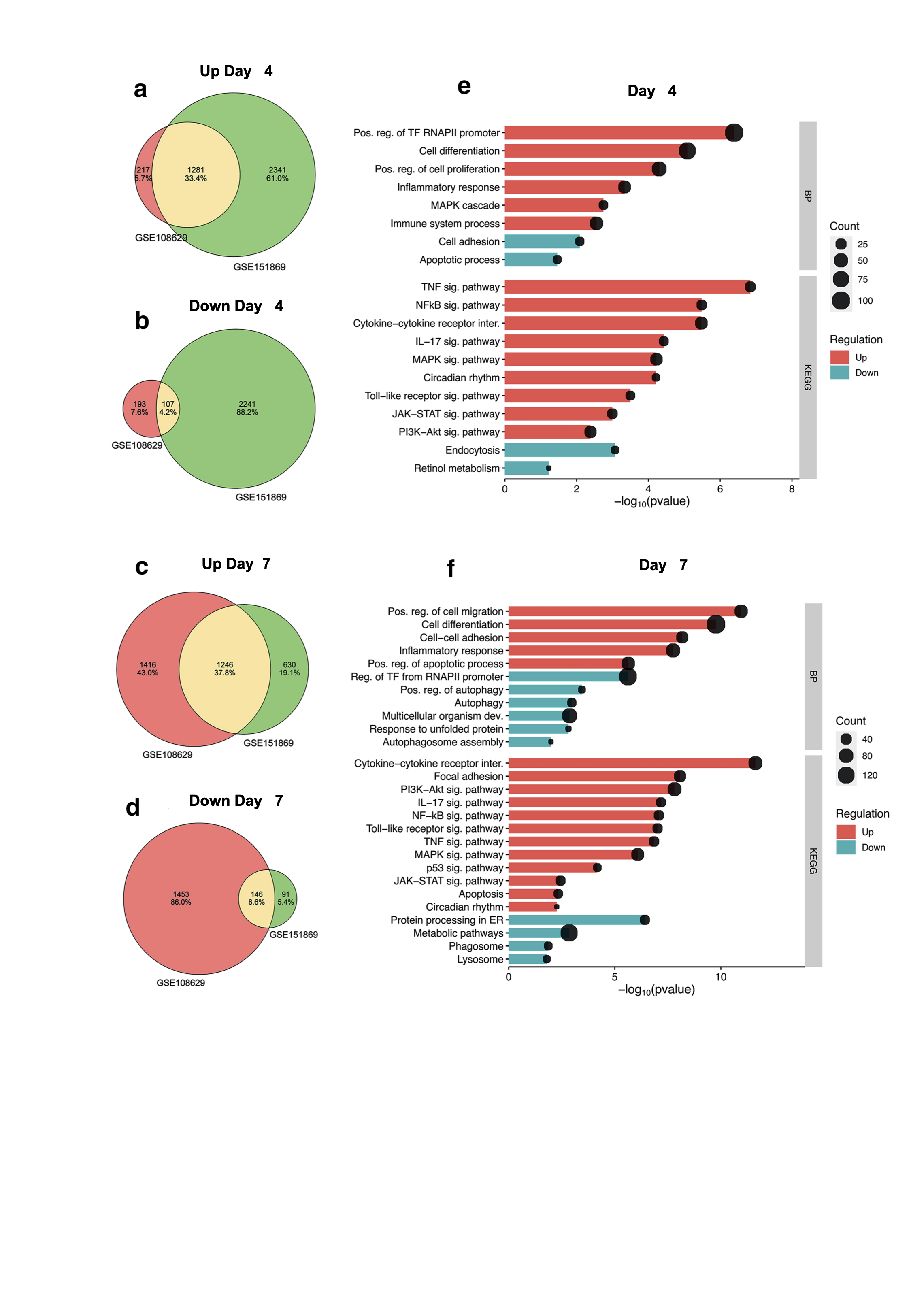

Supplement: S3 Fig — (A–D) Venn diagrams of up- and downregulated DEGs at Day 4 (A–B) and Day 7 (C–D). (E–F) Top enriched GO terms and KEGG pathways. (TIFF) [file pone.0352764.s003.tiff]

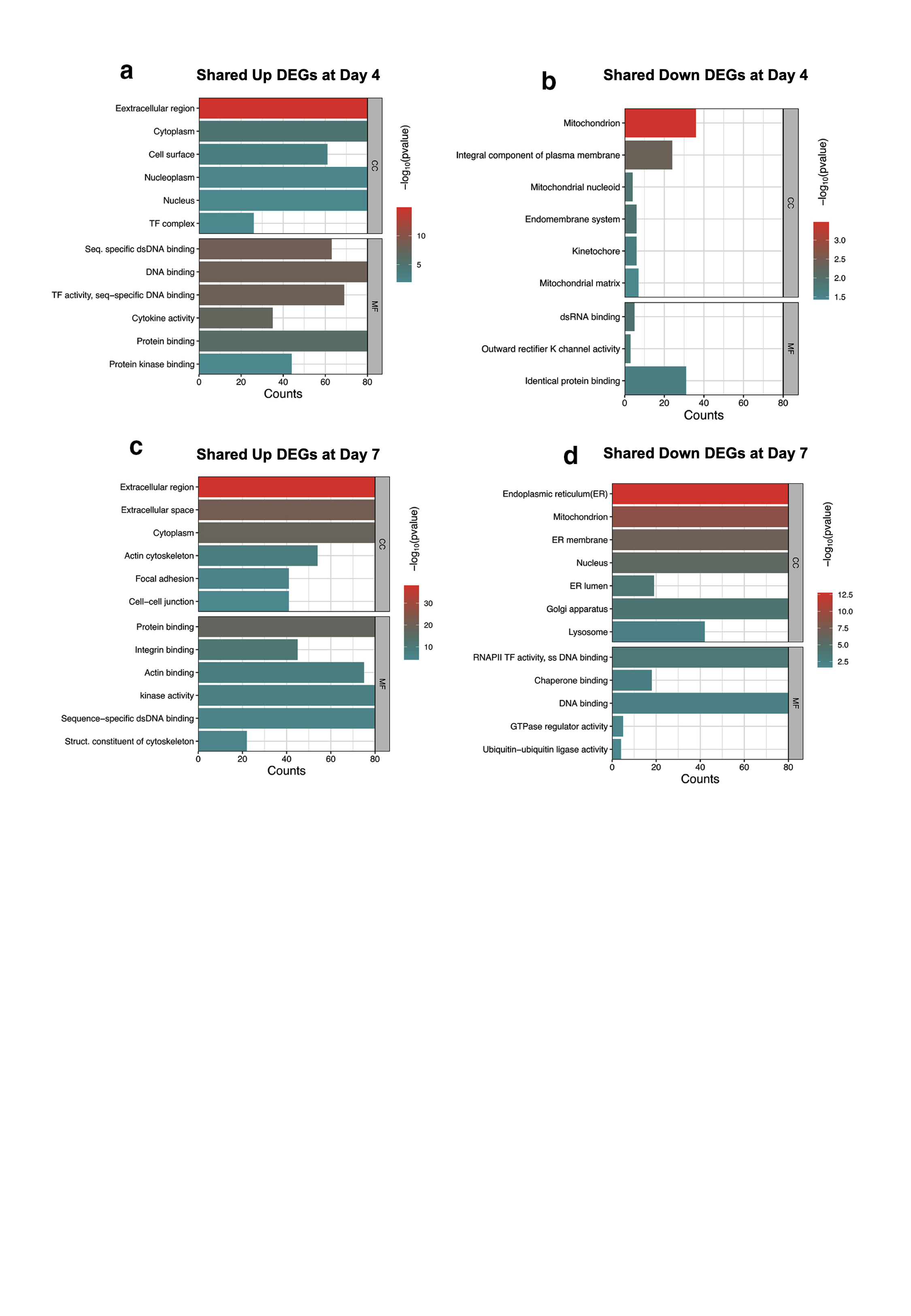

Supplement: S4 Fig — (A–B) CC and MF enrichment of shared DEGs at Day 4. (C–D) CC and MF enrichment of shared DEGs at Day 7. (TIFF) [file pone.0352764.s004.tiff]

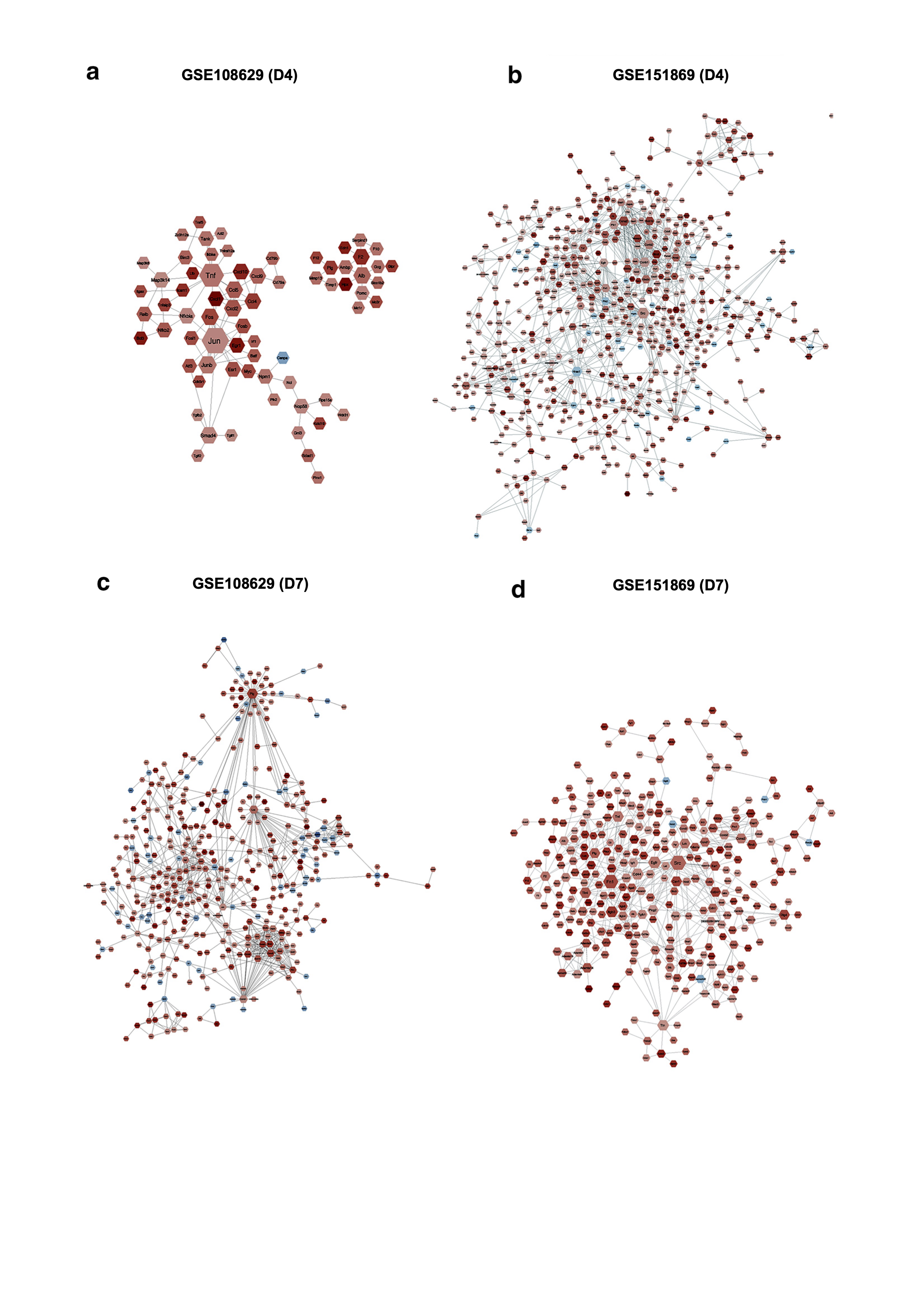

Supplement: S5 Fig — (A–B) PPI networks at Day 4. (C–D) PPI networks at Day 7. Node size and color represent degree and fold-change, respectively. (TIFF) [file pone.0352764.s005.tiff]
