# Supplementary material for: Integrated transcriptomic analysis of LMB2-induced podocyte injury identifies conserved inflammatory and adaptive stress responses
Source: PLoS One. 2026 Jul 31;21(7):e0352764. doi: 10.1371/journal.pone.0352764 (PMC13426963; doi:10.1371/journal.pone.0352764)
Supplement: Attachment — Submitted filename: Response letter.docx [file pone.0352764.s008.docx]

**Reviewer #1:**

This study utilizes two public datasets to perform an integrated time-series transcriptomic analysis of LMB2 induced podocyte injury, revealing a biphasic pattern transitioning from inflammatory responses (day 4) to ER stress/autophagy impairment (day 7), which carries certain clinical relevance. However, several issues remain in the following aspects:

1.In the Abstract, “FSGS” is used, but the full term “focal segmental glomerulosclerosis” appears repeatedly in the main text. It is recommended to use the abbreviation consistently after its first appearance.

We thank the reviewer for this helpful suggestion. We have corrected this inconsistency. The term “focal segmental glomerulosclerosis (FSGS)” is now defined at its first occurrence in the manuscript (Introduction, Paragraph 1), and the abbreviation "FSGS" is used consistently throughout the remainder of the text.

2.All results in this manuscript are derived from re analysis of public data, without independent data validation or experimental verification, which represents the major shortcoming of this work. It is advisable to supplement with validation using an independent dataset or cross validation via Nephroseq / single cell databases.

We thank the reviewer for this critical observation. We agree that de novo experimental validation is an important step in strengthening transcriptomic findings. However, to address this without new wet-lab data, we adopted a multi-step cross-dataset validation strategy to ensure the robustness of our results .

Specifically, we performed differential expression analysis independently in two separate datasets (GSE108629 and GSE151869) and prioritized only those genes and pathways that were reproducibly detected with consistent directionality across both. This approach significantly reduces dataset-specific technical noise and prioritizes conserved biological signals.

Furthermore, in the revised manuscript, we have explicitly acknowledged the lack of external experimental validation as a limitation in the Discussion section (final paragraph): *“These findings should be interpreted within the limitations of a retrospective bulk transcriptomic analysis... Accordingly, these results are hypothesis-generating and require experimental validation in selective podocyte injury models.”* . We believe this rigorous bioinformatic integration provides a reliable foundation for future hypothesis-driven studies.

3.In the Results section, “Early inflammatory responses…” appears on lines 107 123, followed by an identical heading “Sustained inflammatory and stress related responses at day 4” starting at line 124. Additionally, there is a typo “dat 7” that should be “day 7”. These should be corrected.

We sincerely apologize for these editorial oversights and thank the reviewer for their meticulous attention to detail. In response to this comment, we have performed a comprehensive structural and temporal re-evaluation of the manuscript to ensure maximum consistency and professional clarity .

1. Strategic Focus on Comparable Time-points (Day 4 and Day 7): Beyond correcting the redundant headings, we have made the strategic decision to focus the revised manuscript exclusively on Day 4 and Day 7 . The rationale for this refinement is as follows:

- Cross-Dataset Symmetry: While GSE151869 included a Day 1 time point, GSE108629 did not. By removing the Day 1 analysis (previously lines 107-123), we ensure that every stage of our proposed biphasic injury model is supported by convergent evidence from both independent datasets .
- Prioritizing Conserved Signals: This approach eliminates dataset-specific "noise" from the unshared Day 1 time point, strengthening the study’s core focus on transcriptional responses that are reproducibly detected across disparate experimental contexts.

2. Correction of Headings and Typos:

The redundant and incorrect headings have been replaced with descriptive titles that accurately reflect this more robust temporal architecture:

- The heading for the early phase now reads: “A conserved early transcriptional injury response is detected across datasets at Day 4”.
- The heading for the late phase now reads: “A sustained and expanded conserved injury program is detected across datasets at Day 7”.
- The typographical error (“dat 7”) has been corrected to “Day 7” throughout the entire manuscript, including the expanded Methods and Results sections.

We believe this focused analytical framework provides a significantly more coherent and reproducible molecular signature of podocyte injury .

4.The authors state that “DEGs were calculated separately… to avoid batch effects”; please clarify why batch correction was not performed.

We appreciate the opportunity to clarify our methodological rationale regarding batch effects. Traditional batch correction algorithms (such as ComBat) are typically utilized when raw expression matrices from multiple studies are merged into a single meta-matrix for joint statistical modeling. However, merging raw data from independent laboratories can often introduce artificial variance that is difficult to fully adjust without masking subtle biological signals.

To ensure maximum technical rigor, we opted for a more conservative "Independent-then-Intersect" integration strategy . Instead of merging raw data, we performed differential expression analysis independently within each dataset relative to its own internal control group . Cross-dataset integration was achieved only at the final step by intersecting the resulting DEG lists and retaining genes with consistent regulation directionality .

Importantly, as a direct consequence of this more stringent re-analysis and the strategic focus on cross-dataset symmetry, the final number of genes in our "final pooled shared DEG set" has been updated to 725 genes (706 upregulated and 19 downregulated) . This shift reflects a more accurate, robust, and biologically consistent molecular signature compared to the initial submission, as it prioritizes only those signals that survive the strictest independent validation across both studies.

We have expanded the "Data preprocessing and differential expression analysis" section in the Methods to explicitly state this rationale .

5.Immune cell signatures are mentioned, but the methodology is not described and specific changes in cell types are not shown. It is recommended to provide further details.

We thank the reviewer for this insightful comment. We completely agree that immune-cell signatures cannot be reliably inferred from bulk transcriptomic data without a dedicated deconvolution approach or single-cell validation. To avoid over-interpretation and maintain consistency, we have removed all references to "immune-cell signatures" from the Abstract, Methods, and Results sections. We believe this refinement strengthens the manuscript by ensuring that every conclusion remains strictly within the interpretative scope of the presented transcriptomic evidence.

6.Downregulation of autophagy and lysosomal pathways at day 7 is noted, but the negative enrichment of “ESCRT complexes” and “COPII” in the GSEA results is not discussed in depth. Further elaboration is warranted.

We thank the reviewer for this insightful suggestion. We agree that the suppression of ESCRT complexes and COPII-mediated trafficking provides critical insights into the maladaptive state of injured podocytes at Day 7. In response, we have substantially expanded the Discussion to provide a structured, evidence-based interpretation of these findings .

To ensure interpretive robustness, we conducted a targeted re-evaluation of the GSEA leader-edge genes and our shared DEG sets:

- Evidence-Based Selection: While individual datasets showed various COPII-related changes, we prioritized signals supported by convergent evidence. Specifically, we highlighted the marked downregulation of Chmp4b (a core component of the ESCRT-III complex), which was reproducibly detected as a shared DEG across both datasets.
- Functional Integration: In the revised Discussion (Paragraph 6), we now elaborate on how the coordinated downregulation of ESCRT and COPII pathways reflects a fundamental breakdown in vesicular trafficking. This impairment is discussed as a key driver that limits membrane turnover and the clearance of misfolded proteins, thereby exacerbating unresolved chronic ER stress and compromising podocyte structural integrity .
- Connection to Proteostasis: These findings are now integrated into our proposed model of progressive proteostatic imbalance, linking defective intracellular cargo processing to increased apoptotic susceptibility in the late phase of injury .

We believe this deeper elaboration provides a more coherent mechanistic framework for the transition toward podocyte failure at Day 7.

**Reviewer #2:**

This manuscript re-analyzes two public transcriptomic datasets (GSE108629 and GSE151869) to characterize temporal molecular responses in LMB2-induced podocyte injury. The study is potentially useful because it attempts to define a biphasic response pattern, with early inflammatory signaling and later ER stress/autophagy-lysosome dysfunction. The overall analytical direction is relevant to podocyte biology and glomerular disease research. However, in its current form, the manuscript remains largely hypothesis-generating, and several methodological, interpretive, and reporting issues need to be addressed before the work can be considered for publication.

Major comments

1. The manuscript describes LMB2-induced podocyte damage as “a relevant AKI model.” This is not well justified. LMB2 is primarily a selective podocyte injury model relevant to podocytopathy/FSGS-like biology, not a conventional AKI model. The current wording risks misleading readers regarding the biological scope of the model. The introduction should be revised to better distinguish selective podocyte injury from broader AKI paradigms, even if the authors wish to discuss kidney injury progression more generally.

We thank the reviewer for this crucial conceptual correction. We agree that characterizing the LMB2-induced injury as a generalized AKI paradigm was technically inaccurate. In response, we have recalibrated the conceptual framework of the manuscript to align with the specific biological nature of this model.

The following revisions have been implemented:

- Refined Introduction: We have revised the Introduction (Paragraphs 1 and 5) to clearly distinguish selective podocyte injury from broader, non-selective AKI models (e.g., ischemia-reperfusion).
- Terminology Standardization: All instances where the LMB2 model was framed as a generalized AKI model have been removed. The text now consistently describes it as a model of selective podocyte damage relevant to primary glomerular diseases and FSGS-like pathology.
- Textual Clarification: We have added the following statement to the revised Introduction: *“This process is a defining feature of primary glomerular diseases and is distinct from generalized acute kidney injury (AKI)... Accordingly, LMB2-induced injury is generally regarded as a model of selective podocyte damage rather than a broad AKI model.”*.

We believe these revisions ensure that the study’s findings are interpreted within the correct clinical and pathophysiological context.

2. Although the authors provide the general workflow, the methodological description remains incomplete. Important information is missing or insufficiently described, including sample numbers per group/time point, array platforms, exact comparison design, handling of controls at each time point, annotation source/version, GSEA gene set database and parameters, X2K settings, STRING/Cytoscape selection criteria, and the rationale for selecting only the commonly upregulated DEGs for topological prioritization. The statement that results were “synthesized at the pathway level” is too vague to support reproducibility. The Methods should be expanded substantially.

We thank the reviewer for emphasizing the necessity of methodological transparency, which is fundamental to the reproducibility of computational research. In the revised manuscript, we have substantially expanded and restructured the Materials and Methods section to provide a granular and verifiable analytical framework.

The following technical details and strategic refinements have been implemented:

- Enhanced Parametric Detail: We have explicitly defined previously ambiguous parameters, including GSEA metrics (Reactome collection, Signal2Noise), high-confidence interaction thresholds for STRING (score ≥ 0.9), and network topology criteria (ranking nodes by degree and betweenness centrality).
- Visual Roadmap (Figure 1): To provide a clear overview of our pipeline, we have included a comprehensive bioinformatic workflow (Figure 1). This flowchart serves as a visual roadmap, delineating each step from initial data retrieval and independent analysis to the final biological interpretation.
- Methodological Rigor via Data Filtering: To maximize cross-dataset symmetry, we made the strategic decision to focus exclusively on Day 4 and Day 7 time points. By removing Day 1 (which lacked a counterpart in one dataset), we ensure that our "Independent-then-Intersect" strategy is strictly applied to reproducible signals only.
- Transparency of Re-analysis: We have clarified the rationale for analyzing datasets independently relative to their internal controls before integration, an approach that effectively bypasses batch effect artifacts while maintaining biological integrity.

By providing this level of detail and a visual representation of the workflow, we believe we have addressed the previous ambiguities and ensured that our pipeline is fully transparent and reproducible.

3. The Abstract states that “immune-cell signatures” were analyzed, but no clear corresponding method, result, or figure is presented. This should either be removed or properly documented. In the Results section, the heading for the day 7 subsection is incorrectly repeated as “Sustained inflammatory and stress-related responses at day 4,” which suggests insufficient proofreading. There is also inconsistency regarding the key hubs: the Abstract highlights RELA, JUN, ATF4, MAPK14, and ShcC, whereas the Results emphasize SUZ12/REST/TP63 and CDK2/GSK3β, and the Discussion later shifts again to RELA, JUN, ATF4, ShcC, and Cdc42. The manuscript needs a much clearer and more consistent hierarchy of “hub regulators.”

We appreciate the reviewer’s meticulous review of our manuscript’s internal consistency. We have addressed these points by implementing a rigid editorial and scientific audit:

1. Removal of Immune-Cell Claims: We have entirely removed references to "immune-cell signatures" across the Abstract and Results. To maintain technical rigor, we now focus exclusively on pathway-level inflammatory signatures (e.g., TNF/NF-κB signaling) which are directly supported by our shared DEG analysis.
2. Correction of Headings: The duplicated and incorrect heading in the Results has been resolved. Following our strategic focus on comparable time-points, the section now clearly reads: *“A sustained and expanded conserved injury program is detected across datasets at Day 7”*.
3. Unified Regulatory Hierarchy: To resolve inconsistencies, we have harmonized the reporting of key regulators across all sections (Abstract, Results, and Discussion). We now consistently present a three-tier regulatory hierarchy derived from our X2K and network analysis:
   - Transcriptional Hub: RELA (identified as the primary node across both datasets).
   - Epigenetic Modulators: EZH2 and SUZ12 (top-ranked epigenetic regulators).
   - Upstream Kinases: MAPK14 and AKT1.

This structured framework ensures that the regulatory landscape of podocyte injury is presented with absolute consistency and clarity throughout the manuscript.

4. The Discussion frequently moves from transcriptomic enrichment to mechanistic assertions about TLR signaling, TNF-α activity, LTβ, IL-6, IL-8, CSF1, PI3K, JAK-STAT3, immune recruitment, and dedifferentiation. However, the current study design does not directly demonstrate cytokine activity, cell-cell communication, immune infiltration, or functional pathway activation. These points may be reasonable hypotheses, but they should be presented as inferred possibilities rather than established mechanisms. This issue is especially important in a purely computational re-analysis study.

We completely agree with the reviewer that bioinformatic predictions, while powerful, require experimental confirmation to establish definitive causality. In response, we have moderated the tone and language throughout the entire manuscript to align with the exploratory and hypothesis-generating nature of this study.

Key revisions include:

- Hedged Language: We have replaced definitive terms (e.g., "demonstrates," "proves," "causes") with more appropriate academic qualifiers such as "suggests," "is associated with," "potentially mediates," and "points toward".
- Defined Study Scope: We now explicitly characterize our findings as a "foundational bioinformatic framework" intended to prioritize high-confidence targets for future functional studies, rather than a definitive mechanistic proof.
- Expanded Limitations: As also requested by Reviewer #1, we have added a dedicated paragraph to the Discussion emphasizing that while our "Independent-then-Intersect" strategy identifies highly robust and conserved signals (725 shared DEGs), these results should be interpreted as computational predictions that warrant downstream wet-lab validation in podocyte-specific injury models.

We believe this more cautious and balanced interpretive approach accurately reflects the scope of the data while maintaining the scientific value of our predictive findings.

Minor comments

1. The manuscript would benefit from careful language editing. There are several awkward or imprecise phrasings, such as “FSGS, etc.” in the Abstract, “dat 7” in the Methods, and inconsistent wording around signaling interpretations.

We sincerely apologize for the typographical errors and the imprecise phrasing in the original submission. We recognize that such oversights can detract from the clarity of the scientific findings. In response, we have conducted a rigorous linguistic and structural audit of the entire manuscript .

Specific corrections include:

- Refining Terminology: Imprecise expressions such as "FSGS, etc." have been removed . The disease context is now clearly defined as "focal segmental glomerulosclerosis (FSGS)" upon its first mention in the Introduction, with the abbreviation used consistently thereafter.
- Correcting Technical Typos: All typographical errors, including "dat 7," have been corrected to "Day 7" throughout the text, including the expanded Methods and Result sections .
- Harmonizing Signaling Interpretations: We have recalibrated our language regarding molecular signaling to ensure consistency . As noted in our response to Major Comment 4, we have replaced causal assertions with association-based terminology (e.g., "consistent with," "associated with") to accurately reflect the inferential nature of transcriptomic re-analysis .

We believe these revisions have significantly enhanced the readability and professional tone of the manuscript, ensuring that the presentation now matches the technical rigor of the updated methodology.

2. Please define more clearly what is meant by “robustness” of the biphasic model, given that this is based on two public datasets without experimental confirmation.

We thank the reviewer for this important clarification. We agree that the term “robustness” could be misinterpreted as implying de novo experimental validation. To ensure technical precision, we have refined our terminology throughout the manuscript.

Specifically, we have replaced "robustness" with terms such as "conserved," "shared," or "reproducible across datasets". In the context of our study, these terms refer specifically to transcriptomic reproducibility, defined by:

1. Directional Consistency: Identifying differentially expressed genes (DEGs) that show the same direction of regulation (up or down) across independent studies.
2. Cross-Dataset Convergence: Prioritizing molecular pathways and regulatory hubs that are significantly enriched in both GSE108629 and GSE151869 datasets, thereby reducing the influence of dataset-specific technical noise.

We have added a clarifying statement in the Discussion to define this framework : *“By prioritizing shared transcriptional changes, this approach captures core molecular programs underlying podocyte injury.”* Furthermore, we have explicitly stated in the limitation section that this statistical reproducibility is hypothesis-generating and must be distinguished from functional experimental validation .

3. The authors should explicitly state whether all analyses were performed separately in each dataset and then intersected, or whether any merged ranking/statistical integration strategy was used at later steps.

We appreciate this suggestion for methodological clarification. We have explicitly stated our analytical strategy in the revised "Materials and Methods" section under the subheadings "Data preprocessing and differential expression analysis" and "Identification of shared differentially expressed genes" .

Specifically, to ensure maximum technical rigor:

1. Independent Analysis: Differential expression analysis was performed strictly independently within each dataset (GSE108629 and GSE151869) at each post-injury time point relative to its own internal control .
2. Intersection Strategy: Cross-dataset integration was achieved only at the final step by intersecting the resulting DEG lists. Only genes showing consistent regulation directionality across both datasets were retained for downstream functional and network analyses.

We intentionally avoided merged ranking or across-dataset statistical integration of expression values to minimize potential cross-study technical confounding and batch effects that could arise from merging raw data from independent laboratories. This approach prioritizes biologically reproducible signals that are conserved across independent experimental contexts.

4. Figure-to-text consistency should be improved. In particular, the figures and legends should more directly support the claims made about immune-cell signatures, vesicle transport directionality, and hub prioritization.

We thank the reviewer for this helpful comment. We have conducted a comprehensive synchronization between the main text, figure legends, and visual data to ensure perfect alignment and interpretive rigor .

Specifically, we have addressed the three areas identified:

- Immune-Cell Signatures: As noted in our responses to Reviewer #1 (Comment 5) and Reviewer #2 (Major Comment 3), we have entirely removed all unsupported claims regarding immune-cell signatures from the Abstract, Results, and Figure legends to avoid over-interpretation of bulk transcriptomic data .
- Vesicle Transport Directionality: We have refined the text to accurately reflect the directionality shown in the GSEA plots (Fig 2e and 3e). The manuscript now explicitly distinguishes between the negative enrichment (downregulation) of anterograde transport/COPII/ESCRT complexes and the positive enrichment of retrograde ER-to-Golgi transport at Day 7, providing a consistent model of vesicular trafficking disruption .
- Hub Prioritization: We have harmonized the reporting of hub genes and upstream regulators . The hierarchy discussed in the Results and Discussion now directly corresponds to the topological ranking in Table S2 and the visual representation in Figure 5 . This ensures that the RELA-centered inflammatory axis and the supporting epigenetic/kinase layers are presented with a unified and transparent evidence base.

We believe these revisions have resolved all inconsistencies, ensuring that every claim in the manuscript is now directly and transparently supported by the corresponding figures and legends.
