# Supplementary material for: Integrated transcriptomic analysis of LMB2-induced podocyte injury identifies conserved inflammatory and adaptive stress responses
Source: PLoS One. 2026 Jul 31;21(7):e0352764. doi: 10.1371/journal.pone.0352764 (PMC13426963; doi:10.1371/journal.pone.0352764)
Supplement: Attachment — Submitted filename: Response to Reviewer-second time- PLOS ONE.docx [file pone.0352764.s009.docx]

**Date:** June 06, 2026

**To:** Junzheng Yang Academic Editor, PLOS ONE

**Subject:** Response to Reviewers and Submission of Revised Manuscript [PONE-D-26-00699]

Dear Dr. Junzheng Yang,

Thank you for the opportunity to revise and resubmit our manuscript entitled “Integrated transcriptomic analysis of LMB2-induced podocyte injury identifies conserved inflammatory and adaptive stress responses” (PONE-D-26-00699R1).

We are grateful to the editor and reviewers for their constructive evaluation. In this revision, we have addressed the remaining comment regarding the biological relevance of ESCRT and COPII downregulation in podocytes. Specifically, we expanded the Discussion to clarify how COPII-mediated ER export, ESCRT-dependent endosomal cargo sorting, lysosome-directed degradation, and autophagy–lysosome function may contribute to proteostasis maintenance and podocyte structural vulnerability.

Please find enclosed the revised manuscript, a marked-up version with tracked changes, and a point-by-point response to the reviewer comments.

Sincerely yours,

**Reza Moghadasali**

(On behalf of the authors of the manuscript)

Department of Stem Cells and Developmental Biology, Royan Institute for Stem Cell Biology and Technology, Tehran, Iran.

Emails: rezamoghadasali@yahoo.com / s.taleahmad@royan-rc.ac.ir

**Reviewer #1 comment:**

After the previous round of revisions, the quality of the manuscript has been further improved, and only minor adjustments are needed. The discussion regarding “ESCRT and COPII” could be deepened further. Since GSEA has shown enrichment of these pathways, why is the downregulation of ESCRT and COPII complexes detrimental to podocytes? Further elaboration on this point would be beneficial.

**Response:**

We thank the reviewer for this constructive suggestion. We have revised and expanded the Discussion to clarify the biological relevance of the ESCRT- and COPII-related GSEA findings.

We now explain that the observed downregulation/negative enrichment of ESCRT- and COPII-associated gene sets may reflect reduced endosomal and secretory trafficking capacity in injured podocytes. This interpretation is biologically relevant because podocytes are highly specialized, polarized epithelial cells whose filtration-barrier function depends on tightly regulated membrane-protein delivery, surface localization, retrieval, recycling, and degradation to maintain slit-diaphragm organization, foot process architecture, and membrane homeostasis. This rationale is supported by prior podocyte studies showing that disruption of endocytic machinery and altered nephrin/podocin turnover impair podocyte foot process integrity and filtration-barrier maintenance. Mechanistically, ESCRT complexes contribute to sorting internalized, often ubiquitinated membrane cargo into multivesicular bodies and lysosomal routes, whereas COPII complexes mediate ER-to-Golgi export of newly synthesized membrane and secretory proteins, supporting secretory delivery and replacement of proteins that require proper processing and surface localization. Therefore, reduced expression of genes associated with these pathways may be consistent with reduced capacity to coordinate both internalized cargo handling and secretory delivery/replacement of membrane-associated proteins, potentially affecting the turnover, localization, and maintenance of slit-diaphragm-associated proteins such as nephrin and podocin during injury. Future studies should directly test this interpretation by assessing ESCRT/COPII component abundance, vesicular trafficking flux, and slit-diaphragm protein localization or turnover in selective podocyte injury models.

The revised text has been added to the Discussion section. [line 339 – line 363]

Reviewer #2:

Comment:

The author has addressed all the issues I raised in my previous round of revisions, and I think this version is ready for acceptance.

Response:

We sincerely thank the reviewer for the careful evaluation of our revised manuscript and for acknowledging that the previous concerns have been addressed. We greatly appreciate the reviewer’s constructive feedback, which helped improve the clarity and quality of the manuscript.
