# Supplementary material for: Integrated transcriptomic analysis of LMB2-induced podocyte injury identifies conserved inflammatory and adaptive stress responses
Source: PLoS One. 2026 Jul 31;21(7):e0352764. doi: 10.1371/journal.pone.0352764 (PMC13426963; doi:10.1371/journal.pone.0352764)
Supplement: Peer review history

# Peer review: Decision Letter 0

**DOI:** 10.1371/journal.pone.0352764.r001

**Authors:** Junzheng Yang

23 Mar 2026

PONE-D-26-00699

Integrated transcriptomic analysis of LMB2-induced podocyte injury identifies conserved inflammatory and adaptive stress responses

PLOS One

Dear Dr. Moghadasali,

Thank you for submitting your manuscript to PLOS ONE. After careful consideration, we feel that it has merit but does not fully meet PLOS ONE’s publication criteria as it currently stands. Therefore, we invite you to submit a revised version of the manuscript that addresses the points raised during the review process.

Please submit your revised manuscript by May 07 2026 11:59PM. If you will need more time than this to complete your revisions, please reply to this message or contact the journal office at plosone@plos.org. When you're ready to submit your revision, log on to https://www.editorialmanager.com/pone/ and select the 'Submissions Needing Revision' folder to locate your manuscript file.

Please include the following items when submitting your revised manuscript:

- A letter that responds to each point raised by the academic editor and reviewer(s). You should upload this letter as a separate file labeled 'Response to Reviewers'.
- A marked-up copy of your manuscript that highlights changes made to the original version. You should upload this as a separate file labeled 'Revised Manuscript with Track Changes'.
- An unmarked version of your revised paper without tracked changes. You should upload this as a separate file labeled 'Manuscript'.

If you would like to make changes to your financial disclosure, please include your updated statement in your cover letter. Guidelines for resubmitting your figure files are available below the reviewer comments at the end of this letter.

If applicable, we recommend that you deposit your laboratory protocols in protocols.io to enhance the reproducibility of your results. Protocols.io assigns your protocol its own identifier (DOI) so that it can be cited independently in the future. For instructions see: https://journals.plos.org/plosone/s/submission-guidelines#loc-laboratory-protocols. Additionally, PLOS ONE offers an option for publishing peer-reviewed Lab Protocol articles, which describe protocols hosted on protocols.io. Read more information on sharing protocols at https://plos.org/protocols?utm_medium=editorial-email&utm_source=authorletters&utm_campaign=protocols.

We look forward to receiving your revised manuscript.

Kind regards,

Junzheng Yang

Academic Editor

PLOS One

Journal Requirements:

When submitting your revision, we need you to address these additional requirements.

1. Please ensure that your manuscript meets PLOS ONE's style requirements, including those for file naming. The PLOS ONE style templates can be found at

https://journals.plos.org/plosone/s/file?id=wjVg/PLOSOne_formatting_sample_main_body.pdf and

https://journals.plos.org/plosone/s/file?id=ba62/PLOSOne_formatting_sample_title_authors_affiliations.pdf

2. PLOS requires an ORCID iD for the corresponding author in Editorial Manager on papers submitted after December 6th, 2016. Please ensure that you have an ORCID iD and that it is validated in Editorial Manager. To do this, go to ‘Update my Information’ (in the upper left-hand corner of the main menu), and click on the Fetch/Validate link next to the ORCID field. This will take you to the ORCID site and allow you to create a new iD or authenticate a pre-existing iD in Editorial Manager.

3. Please note that PLOS One has specific guidelines on code sharing for submissions in which author-generated code underpins the findings in the manuscript. In these cases, we expect all author-generated code to be made available without restrictions upon publication of the work. Please review our guidelines at https://journals.plos.org/plosone/s/materials-and-software-sharing#loc-sharing-code and ensure that your code is shared in a way that follows best practice and facilitates reproducibility and reuse.

4. Please note that your Data Availability Statement is currently missing [the repository name and/or the DOI/accession number of each dataset OR a direct link to access each database]. If your manuscript is accepted for publication, you will be asked to provide these details on a very short timeline. We therefore suggest that you provide this information now, though we will not hold up the peer review process if you are unable.

5. If the reviewer comments include a recommendation to cite specific previously published works, please review and evaluate these publications to determine whether they are relevant and should be cited. There is no requirement to cite these works unless the editor has indicated otherwise.

[Note: HTML markup is below. Please do not edit.]

Reviewers' comments:

Reviewer's Responses to Questions

**Comments to the Author**

1. Is the manuscript technically sound, and do the data support the conclusions?

The manuscript must describe a technically sound piece of scientific research with data that supports the conclusions. Experiments must have been conducted rigorously, with appropriate controls, replication, and sample sizes. The conclusions must be drawn appropriately based on the data presented.

Reviewer #1: Yes

Reviewer #2: No

**********

2. Has the statistical analysis been performed appropriately and rigorously?

Reviewer #1: Yes

Reviewer #2: No

**********

3. Have the authors made all data underlying the findings in their manuscript fully available?

The PLOS Data policy requires authors to make all data underlying the findings described in their manuscript fully available without restriction, with rare exception (please refer to the Data Availability Statement in the manuscript PDF file). The data should be provided as part of the manuscript or its supporting information, or deposited to a public repository. For example, in addition to summary statistics, the data points behind means, medians and variance measures should be available. If there are restrictions on publicly sharing data—e.g. participant privacy or use of data from a third party—those must be specified.

Reviewer #1: Yes

Reviewer #2: Yes

**********

4. Is the manuscript presented in an intelligible fashion and written in standard English?

PLOS ONE does not copyedit accepted manuscripts, so the language in submitted articles must be clear, correct, and unambiguous. Any typographical or grammatical errors should be corrected at revision, so please note any specific errors here.

Reviewer #1: Yes

Reviewer #2: No

**********

5. Review Comments to the Author

Please use the space provided to explain your answers to the questions above. You may also include additional comments for the author, including concerns about dual publication, research ethics, or publication ethics. (Please upload your review as an attachment if it exceeds 20,000 characters)

Reviewer #1: This study utilizes two public datasets to perform an integrated time-series transcriptomic analysis of LMB2‑induced podocyte injury, revealing a biphasic pattern transitioning from inflammatory responses (day 4) to ER stress/autophagy impairment (day 7), which carries certain clinical relevance. However, several issues remain in the following aspects:

1.In the Abstract, “FSGS” is used, but the full term “focal segmental glomerulosclerosis” appears repeatedly in the main text. It is recommended to use the abbreviation consistently after its first appearance.

2.All results in this manuscript are derived from re‑analysis of public data, without independent data validation or experimental verification, which represents the major shortcoming of this work. It is advisable to supplement with validation using an independent dataset or cross‑validation via Nephroseq / single‑cell databases.

3.In the Results section, “Early inflammatory responses…” appears on lines 107‑123, followed by an identical heading “Sustained inflammatory and stress‑related responses at day 4” starting at line 124. Additionally, there is a typo “dat 7” that should be “day 7”. These should be corrected.

4.The authors state that “DEGs were calculated separately… to avoid batch effects”; please clarify why batch correction was not performed.

5.Immune‑cell signatures are mentioned, but the methodology is not described and specific changes in cell types are not shown. It is recommended to provide further details.

6.Downregulation of autophagy and lysosomal pathways at day 7 is noted, but the negative enrichment of “ESCRT complexes” and “COPII” in the GSEA results is not discussed in depth. Further elaboration is warranted.

Reviewer #2: This manuscript re-analyzes two public transcriptomic datasets (GSE108629 and GSE151869) to characterize temporal molecular responses in LMB2-induced podocyte injury. The study is potentially useful because it attempts to define a biphasic response pattern, with early inflammatory signaling and later ER stress/autophagy-lysosome dysfunction. The overall analytical direction is relevant to podocyte biology and glomerular disease research. However, in its current form, the manuscript remains largely hypothesis-generating, and several methodological, interpretive, and reporting issues need to be addressed before the work can be considered for publication.

Major comments

1. The manuscript describes LMB2-induced podocyte damage as “a relevant AKI model.” This is not well justified. LMB2 is primarily a selective podocyte injury model relevant to podocytopathy/FSGS-like biology, not a conventional AKI model. The current wording risks misleading readers regarding the biological scope of the model. The introduction should be revised to better distinguish selective podocyte injury from broader AKI paradigms, even if the authors wish to discuss kidney injury progression more generally.

2. Although the authors provide the general workflow, the methodological description remains incomplete. Important information is missing or insufficiently described, including sample numbers per group/time point, array platforms, exact comparison design, handling of controls at each time point, annotation source/version, GSEA gene set database and parameters, X2K settings, STRING/Cytoscape selection criteria, and the rationale for selecting only the commonly upregulated DEGs for topological prioritization. The statement that results were “synthesized at the pathway level” is too vague to support reproducibility. The Methods should be expanded substantially.

3. The Abstract states that “immune-cell signatures” were analyzed, but no clear corresponding method, result, or figure is presented. This should either be removed or properly documented. In the Results section, the heading for the day 7 subsection is incorrectly repeated as “Sustained inflammatory and stress-related responses at day 4,” which suggests insufficient proofreading. There is also inconsistency regarding the key hubs: the Abstract highlights RELA, JUN, ATF4, MAPK14, and ShcC, whereas the Results emphasize SUZ12/REST/TP63 and CDK2/GSK3β, and the Discussion later shifts again to RELA, JUN, ATF4, ShcC, and Cdc42. The manuscript needs a much clearer and more consistent hierarchy of “hub regulators.”

4. The Discussion frequently moves from transcriptomic enrichment to mechanistic assertions about TLR signaling, TNF-α activity, LTβ, IL-6, IL-8, CSF1, PI3K, JAK-STAT3, immune recruitment, and dedifferentiation. However, the current study design does not directly demonstrate cytokine activity, cell-cell communication, immune infiltration, or functional pathway activation. These points may be reasonable hypotheses, but they should be presented as inferred possibilities rather than established mechanisms. This issue is especially important in a purely computational re-analysis study.

Minor comments

1. The manuscript would benefit from careful language editing. There are several awkward or imprecise phrasings, such as “FSGS, etc.” in the Abstract, “dat 7” in the Methods, and inconsistent wording around signaling interpretations.

2. Please define more clearly what is meant by “robustness” of the biphasic model, given that this is based on two public datasets without experimental confirmation.

3. The authors should explicitly state whether all analyses were performed separately in each dataset and then intersected, or whether any merged ranking/statistical integration strategy was used at later steps.

4. Figure-to-text consistency should be improved. In particular, the figures and legends should more directly support the claims made about immune-cell signatures, vesicle transport directionality, and hub prioritization.

**********

6. PLOS authors have the option to publish the peer review history of their article (what does this mean?). If published, this will include your full peer review and any attached files.

If you choose “no”, your identity will remain anonymous but your review may still be made public.

**Do you want your identity to be public for this peer review?**  For information about this choice, including consent withdrawal, please see our Privacy Policy.

Reviewer #1: **Yes:**  Zhui Ke

Reviewer #2: No

**********

[NOTE: If reviewer comments were submitted as an attachment file, they will be attached to this email and accessible via the submission site. Please log into your account, locate the manuscript record, and check for the action link "View Attachments". If this link does not appear, there are no attachment files.]

To ensure your figures meet our technical requirements, please review our figure guidelines: https://journals.plos.org/plosone/s/figures

You may also use PLOS’s free figure tool, NAAS, to help you prepare publication quality figures: https://journals.plos.org/plosone/s/figures#loc-tools-for-figure-preparation.

NAAS will assess whether your figures meet our technical requirements by comparing each figure against our figure specifications.

# Author comment: Author response to Decision Letter 1

**DOI:** 10.1371/journal.pone.0352764.r002

1 May 2026

Reviewer #1:

This study utilizes two public datasets to perform an integrated time-series transcriptomic analysis of LMB2 induced podocyte injury, revealing a biphasic pattern transitioning from inflammatory responses (day 4) to ER stress/autophagy impairment (day 7), which carries certain clinical relevance. However, several issues remain in the following aspects:

1.In the Abstract, “FSGS” is used, but the full term “focal segmental glomerulosclerosis” appears repeatedly in the main text. It is recommended to use the abbreviation consistently after its first appearance.

We thank the reviewer for this helpful suggestion. We have corrected this inconsistency. The term “focal segmental glomerulosclerosis (FSGS)” is now defined at its first occurrence in the manuscript (Introduction, Paragraph 1), and the abbreviation "FSGS" is used consistently throughout the remainder of the text.

2.All results in this manuscript are derived from re analysis of public data, without independent data validation or experimental verification, which represents the major shortcoming of this work. It is advisable to supplement with validation using an independent dataset or cross validation via Nephroseq / single cell databases.

We thank the reviewer for this critical observation. We agree that de novo experimental validation is an important step in strengthening transcriptomic findings. However, to address this without new wet-lab data, we adopted a multi-step cross-dataset validation strategy to ensure the robustness of our results .

Specifically, we performed differential expression analysis independently in two separate datasets (GSE108629 and GSE151869) and prioritized only those genes and pathways that were reproducibly detected with consistent directionality across both. This approach significantly reduces dataset-specific technical noise and prioritizes conserved biological signals.

Furthermore, in the revised manuscript, we have explicitly acknowledged the lack of external experimental validation as a limitation in the Discussion section (final paragraph): “These findings should be interpreted within the limitations of a retrospective bulk transcriptomic analysis... Accordingly, these results are hypothesis-generating and require experimental validation in selective podocyte injury models.” . We believe this rigorous bioinformatic integration provides a reliable foundation for future hypothesis-driven studies.

3.In the Results section, “Early inflammatory responses…” appears on lines 107 123, followed by an identical heading “Sustained inflammatory and stress related responses at day 4” starting at line 124. Additionally, there is a typo “dat 7” that should be “day 7”. These should be corrected.

We sincerely apologize for these editorial oversights and thank the reviewer for their meticulous attention to detail. In response to this comment, we have performed a comprehensive structural and temporal re-evaluation of the manuscript to ensure maximum consistency and professional clarity .

1. Strategic Focus on Comparable Time-points (Day 4 and Day 7): Beyond correcting the redundant headings, we have made the strategic decision to focus the revised manuscript exclusively on Day 4 and Day 7 . The rationale for this refinement is as follows:

• Cross-Dataset Symmetry: While GSE151869 included a Day 1 time point, GSE108629 did not. By removing the Day 1 analysis (previously lines 107-123), we ensure that every stage of our proposed biphasic injury model is supported by convergent evidence from both independent datasets .

• Prioritizing Conserved Signals: This approach eliminates dataset-specific "noise" from the unshared Day 1 time point, strengthening the study’s core focus on transcriptional responses that are reproducibly detected across disparate experimental contexts.

2. Correction of Headings and Typos:

The redundant and incorrect headings have been replaced with descriptive titles that accurately reflect this more robust temporal architecture:

• The heading for the early phase now reads: “A conserved early transcriptional injury response is detected across datasets at Day 4”.

• The heading for the late phase now reads: “A sustained and expanded conserved injury program is detected across datasets at Day 7”.

• The typographical error (“dat 7”) has been corrected to “Day 7” throughout the entire manuscript, including the expanded Methods and Results sections.

We believe this focused analytical framework provides a significantly more coherent and reproducible molecular signature of podocyte injury .

4.The authors state that “DEGs were calculated separately… to avoid batch effects”; please clarify why batch correction was not performed.

We appreciate the opportunity to clarify our methodological rationale regarding batch effects. Traditional batch correction algorithms (such as ComBat) are typically utilized when raw expression matrices from multiple studies are merged into a single meta-matrix for joint statistical modeling. However, merging raw data from independent laboratories can often introduce artificial variance that is difficult to fully adjust without masking subtle biological signals.

To ensure maximum technical rigor, we opted for a more conservative "Independent-then-Intersect" integration strategy . Instead of merging raw data, we performed differential expression analysis independently within each dataset relative to its own internal control group . Cross-dataset integration was achieved only at the final step by intersecting the resulting DEG lists and retaining genes with consistent regulation directionality .

Importantly, as a direct consequence of this more stringent re-analysis and the strategic focus on cross-dataset symmetry, the final number of genes in our "final pooled shared DEG set" has been updated to 725 genes (706 upregulated and 19 downregulated) . This shift reflects a more accurate, robust, and biologically consistent molecular signature compared to the initial submission, as it prioritizes only those signals that survive the strictest independent validation across both studies.

We have expanded the "Data preprocessing and differential expression analysis" section in the Methods to explicitly state this rationale .

5.Immune cell signatures are mentioned, but the methodology is not described and specific changes in cell types are not shown. It is recommended to provide further details.

We thank the reviewer for this insightful comment. We completely agree that immune-cell signatures cannot be reliably inferred from bulk transcriptomic data without a dedicated deconvolution approach or single-cell validation. To avoid over-interpretation and maintain consistency, we have removed all references to "immune-cell signatures" from the Abstract, Methods, and Results sections. We believe this refinement strengthens the manuscript by ensuring that every conclusion remains strictly within the interpretative scope of the presented transcriptomic evidence.

6.Downregulation of autophagy and lysosomal pathways at day 7 is noted, but the negative enrichment of “ESCRT complexes” and “COPII” in the GSEA results is not discussed in depth. Further elaboration is warranted.

We thank the reviewer for this insightful suggestion. We agree that the suppression of ESCRT complexes and COPII-mediated trafficking provides critical insights into the maladaptive state of injured podocytes at Day 7. In response, we have substantially expanded the Discussion to provide a structured, evidence-based interpretation of these findings .

To ensure interpretive robustness, we conducted a targeted re-evaluation of the GSEA leader-edge genes and our shared DEG sets:

• Evidence-Based Selection: While individual datasets showed various COPII-related changes, we prioritized signals supported by convergent evidence. Specifically, we highlighted the marked downregulation of Chmp4b (a core component of the ESCRT-III complex), which was reproducibly detected as a shared DEG across both datasets.

• Functional Integration: In the revised Discussion (Paragraph 6), we now elaborate on how the coordinated downregulation of ESCRT and COPII pathways reflects a fundamental breakdown in vesicular trafficking. This impairment is discussed as a key driver that limits membrane turnover and the clearance of misfolded proteins, thereby exacerbating unresolved chronic ER stress and compromising podocyte structural integrity .

• Connection to Proteostasis: These findings are now integrated into our proposed model of progressive proteostatic imbalance, linking defective intracellular cargo processing to increased apoptotic susceptibility in the late phase of injury .

We believe this deeper elaboration provides a more coherent mechanistic framework for the transition toward podocyte failure at Day 7.

Reviewer #2:

This manuscript re-analyzes two public transcriptomic datasets (GSE108629 and GSE151869) to characterize temporal molecular responses in LMB2-induced podocyte injury. The study is potentially useful because it attempts to define a biphasic response pattern, with early inflammatory signaling and later ER stress/autophagy-lysosome dysfunction. The overall analytical direction is relevant to podocyte biology and glomerular disease research. However, in its current form, the manuscript remains largely hypothesis-generating, and several methodological, interpretive, and reporting issues need to be addressed before the work can be considered for publication.

Major comments

1. The manuscript describes LMB2-induced podocyte damage as “a relevant AKI model.” This is not well justified. LMB2 is primarily a selective podocyte injury model relevant to podocytopathy/FSGS-like biology, not a conventional AKI model. The current wording risks misleading readers regarding the biological scope of the model. The introduction should be revised to better distinguish selective podocyte injury from broader AKI paradigms, even if the authors wish to discuss kidney injury progression more generally.

We thank the reviewer for this crucial conceptual correction. We agree that characterizing the LMB2-induced injury as a generalized AKI paradigm was technically inaccurate. In response, we have recalibrated the conceptual framework of the manuscript to align with the specific biological nature of this model.

The following revisions have been implemented:

• Refined Introduction: We have revised the Introduction (Paragraphs 1 and 5) to clearly distinguish selective podocyte injury from broader, non-selective AKI models (e.g., ischemia-reperfusion).

• Terminology Standardization: All instances where the LMB2 model was framed as a generalized AKI model have been removed. The text now consistently describes it as a model of selective podocyte damage relevant to primary glomerular diseases and FSGS-like pathology.

• Textual Clarification: We have added the following statement to the revised Introduction: “This process is a defining feature of primary glomerular diseases and is distinct from generalized acute kidney injury (AKI)... Accordingly, LMB2-induced injury is generally regarded as a model of selective podocyte damage rather than a broad AKI model.”.

We believe these revisions ensure that the study’s findings are interpreted within the correct clinical and pathophysiological context.

2. Although the authors provide the general workflow, the methodological description remains incomplete. Important information is missing or insufficiently described, including sample numbers per group/time point, array platforms, exact comparison design, handling of controls at each time point, annotation source/version, GSEA gene set database and parameters, X2K settings, STRING/Cytoscape selection criteria, and the rationale for selecting only the commonly upregulated DEGs for topological prioritization. The statement that results were “synthesized at the pathway level” is too vague to support reproducibility. The Methods should be expanded substantially.

We thank the reviewer for emphasizing the necessity of methodological transparency, which is fundamental to the reproducibility of computational research. In the revised manuscript, we have substantially expanded and restructured the Materials and Methods section to provide a granular and verifiable analytical framework.

The following technical details and strategic refinements have been implemented:

• Enhanced Parametric Detail: We have explicitly defined previously ambiguous parameters, including GSEA metrics (Reactome collection, Signal2Noise), high-confidence interaction thresholds for STRING (score ≥ 0.9), and network topology criteria (ranking nodes by degree and betweenness centrality).

• Visual Roadmap (Figure 1): To provide a clear overview of our pipeline, we have included a comprehensive bioinformatic workflow (Figure 1). This flowchart serves as a visual roadmap, delineating each step from initial data retrieval and independent analysis to the final biological interpretation.

• Methodological Rigor via Data Filtering: To maximize cross-dataset symmetry, we made the strategic decision to focus exclusively on Day 4 and Day 7 time points. By removing Day 1 (which lacked a counterpart in one dataset), we ensure that our "Independent-then-Intersect" strategy is strictly applied to reproducible signals only.

• Transparency of Re-analysis: We have clarified the rationale for analyzing datasets independently relative to their internal controls before integration, an approach that effectively bypasses batch effect artifacts while maintaining biological integrity.

By providing this level of detail and a visual representation of the workflow, we believe we have addressed the previous ambiguities and ensured that our pipeline is fully transparent and reproducible.

3. The Abstract states that “immune-cell signatures” were analyzed, but no clear corresponding method, result, or figure is presented. This should either be removed or properly documented. In the Results section, the heading for the day 7 subsection is incorrectly repeated as “Sustained inflammatory and stress-related responses at day 4,” which suggests insufficient proofreading. There is also inconsistency regarding the key hubs: the Abstract highlights RELA, JUN, ATF4, MAPK14, and ShcC, whereas the Results emphasize SUZ12/REST/TP63 and CDK2/GSK3β, and the Discussion later shifts again to RELA, JUN, ATF4, ShcC, and Cdc42. The manuscript needs a much clearer and more consistent hierarchy of “hub regulators.”

We appreciate the reviewer’s meticulous review of our manuscript’s internal consistency. We have addressed these points by implementing a rigid editorial and scientific audit:

1. Removal of Immune-Cell Claims: We have entirely removed references to "immune-cell signatures" across the Abstract and Results. To maintain technical rigor, we now focus exclusively on pathway-level inflammatory signatures (e.g., TNF/NF-κB signaling) which are directly supported by our shared DEG analysis.

2. Correction of Headings: The duplicated and incorrect heading in the Results has been resolved. Following our strategic focus on comparable time-points, the section now clearly reads: “A sustained and expanded conserved injury program is detected across datasets at Day 7”.

3. Unified Regulatory Hierarchy: To resolve inconsistencies, we have harmonized the reporting of key regulators across all sections (Abstract, Results, and Discussion). We now consistently present a three-tier regulatory hierarchy derived from our X2K and network analysis:

o Transcriptional Hub: RELA (identified as the primary node across both datasets).

o Epigenetic Modulators: EZH2 and SUZ12 (top-ranked epigenetic regulators).

o Upstream Kinases: MAPK14 and AKT1.

This structured framework ensures that the regulatory landscape of podocyte injury is presented with absolute consistency and clarity throughout the manuscript.

4. The Discussion frequently moves from transcriptomic enrichment to mechanistic assertions about TLR signaling, TNF-α activity, LTβ, IL-6, IL-8, CSF1, PI3K, JAK-STAT3, immune recruitment, and dedifferentiation. However, the current study design does not directly demonstrate cytokine activity, cell-cell communication, immune infiltration, or functional pathway activation. The

Attachment Submitted filename: Response letter.docx

# Peer review: Decision Letter 1

**DOI:** 10.1371/journal.pone.0352764.r003

**Authors:** Junzheng Yang

27 May 2026

PONE-D-26-00699R1

Integrated transcriptomic analysis of LMB2-induced podocyte injury identifies conserved inflammatory and adaptive stress responses

PLOS One

Dear Dr. Moghadasali,

Thank you for submitting your manuscript to PLOS ONE. After careful consideration, we feel that it has merit but does not fully meet PLOS ONE’s publication criteria as it currently stands. Therefore, we invite you to submit a revised version of the manuscript that addresses the points raised during the review process.

Please submit your revised manuscript by Jul 11 2026 11:59PM. If you will need more time than this to complete your revisions, please reply to this message or contact the journal office at plosone@plos.org. When you're ready to submit your revision, log on to https://www.editorialmanager.com/pone/ and select the 'Submissions Needing Revision' folder to locate your manuscript file.

Please include the following items when submitting your revised manuscript:

- A letter that responds to each point raised by the academic editor and reviewer(s). You should upload this letter as a separate file labeled 'Response to Reviewers'.
- A marked-up copy of your manuscript that highlights changes made to the original version. You should upload this as a separate file labeled 'Revised Manuscript with Track Changes'.
- An unmarked version of your revised paper without tracked changes. You should upload this as a separate file labeled 'Manuscript'.

If you would like to make changes to your financial disclosure, please include your updated statement in your cover letter. Guidelines for resubmitting your figure files are available below the reviewer comments at the end of this letter.

If applicable, we recommend that you deposit your laboratory protocols in protocols.io to enhance the reproducibility of your results. Protocols.io assigns your protocol its own identifier (DOI) so that it can be cited independently in the future. For instructions see: https://journals.plos.org/plosone/s/submission-guidelines#loc-laboratory-protocols. Additionally, PLOS ONE offers an option for publishing peer-reviewed Lab Protocol articles, which describe protocols hosted on protocols.io. Read more information on sharing protocols at https://plos.org/protocols?utm_medium=editorial-email&utm_source=authorletters&utm_campaign=protocols.

As the corresponding author, your ORCID iD is verified in the submission system and will appear in the published article. PLOS supports the use of ORCID, and we encourage all coauthors to register for an ORCID iD and use it as well. Please encourage your coauthors to verify their ORCID iD within the submission system before final acceptance, as unverified ORCID iDs will not appear in the published article. *Only*  the individual author can complete the verification step; PLOS staff *cannot*  verify ORCID iDs on behalf of authors.

We look forward to receiving your revised manuscript.

Kind regards,

Junzheng Yang

Academic Editor

PLOS One

Journal Requirements:

If the reviewer comments include a recommendation to cite specific previously published works, please review and evaluate these publications to determine whether they are relevant and should be cited. There is no requirement to cite these works unless the editor has indicated otherwise.

Please review your reference list to ensure that it is complete and correct. If you have cited papers that have been retracted, please include the rationale for doing so in the manuscript text, or remove these references and replace them with relevant current references. Any changes to the reference list should be mentioned in the rebuttal letter that accompanies your revised manuscript. If you need to cite a retracted article, indicate the article’s retracted status in the References list and also include a citation and full reference for the retraction notice.

[Note: HTML markup is below. Please do not edit.]

Reviewers' comments:

Reviewer's Responses to Questions

**Comments to the Author**

1. If the authors have adequately addressed your comments raised in a previous round of review and you feel that this manuscript is now acceptable for publication, you may indicate that here to bypass the “Comments to the Author” section, enter your conflict of interest statement in the “Confidential to Editor” section, and submit your "Accept" recommendation.

Reviewer #1: All comments have been addressed

Reviewer #2: (No Response)

**********

2. Is the manuscript technically sound, and do the data support the conclusions?

The manuscript must describe a technically sound piece of scientific research with data that supports the conclusions. Experiments must have been conducted rigorously, with appropriate controls, replication, and sample sizes. The conclusions must be drawn appropriately based on the data presented.

Reviewer #1: Yes

Reviewer #2: (No Response)

**********

3. Has the statistical analysis been performed appropriately and rigorously?

Reviewer #1: Yes

Reviewer #2: (No Response)

**********

4. Have the authors made all data underlying the findings in their manuscript fully available?

The PLOS Data policy requires authors to make all data underlying the findings described in their manuscript fully available without restriction, with rare exception (please refer to the Data Availability Statement in the manuscript PDF file). The data should be provided as part of the manuscript or its supporting information, or deposited to a public repository. For example, in addition to summary statistics, the data points behind means, medians and variance measures should be available. If there are restrictions on publicly sharing data—e.g. participant privacy or use of data from a third party—those must be specified.

Reviewer #1: Yes

Reviewer #2: (No Response)

**********

5. Is the manuscript presented in an intelligible fashion and written in standard English?

PLOS ONE does not copyedit accepted manuscripts, so the language in submitted articles must be clear, correct, and unambiguous. Any typographical or grammatical errors should be corrected at revision, so please note any specific errors here.

Reviewer #1: Yes

Reviewer #2: (No Response)

**********

6. Review Comments to the Author

Please use the space provided to explain your answers to the questions above. You may also include additional comments for the author, including concerns about dual publication, research ethics, or publication ethics. (Please upload your review as an attachment if it exceeds 20,000 characters)

Reviewer #1: After the previous round of revisions, the quality of the manuscript has been further improved, and only minor adjustments are needed. The discussion regarding "ESCRT and COPII" could be deepened further. Since GSEA has shown enrichment of these pathways, why is the downregulation of ESCRT and COPII complexes detrimental to podocytes? Further elaboration on this point would be beneficial.

Reviewer #2: The author has addressed all the issues I raised in my previous round of revisions, and I think this version is ready for acceptance.

**********

7. PLOS authors have the option to publish the peer review history of their article (what does this mean?). If published, this will include your full peer review and any attached files.

If you choose “no”, your identity will remain anonymous but your review may still be made public.

**Do you want your identity to be public for this peer review?**  For information about this choice, including consent withdrawal, please see our Privacy Policy.

Reviewer #1: **Yes:**  Zhui Ke

Reviewer #2: No

**********

[NOTE: If reviewer comments were submitted as an attachment file, they will be attached to this email and accessible via the submission site. Please log into your account, locate the manuscript record, and check for the action link "View Attachments". If this link does not appear, there are no attachment files.]

To ensure your figures meet our technical requirements, please review our figure guidelines: https://journals.plos.org/plosone/s/figures

You may also use PLOS’s free figure tool, NAAS, to help you prepare publication quality figures: https://journals.plos.org/plosone/s/figures#loc-tools-for-figure-preparation.

NAAS will assess whether your figures meet our technical requirements by comparing each figure against our figure specifications.

# Author comment: Author response to Decision Letter 2

**DOI:** 10.1371/journal.pone.0352764.r004

5 Jun 2026

Date: June 06, 2026

To: Junzheng Yang Academic Editor, PLOS ONE

Subject: Response to Reviewers and Submission of Revised Manuscript [PONE-D-26-00699]

Dear Dr. Junzheng Yang,

Thank you for the opportunity to revise and resubmit our manuscript entitled “Integrated transcriptomic analysis of LMB2-induced podocyte injury identifies conserved inflammatory and adaptive stress responses” (PONE-D-26-00699R1).

We are grateful to the editor and reviewers for their constructive evaluation. In this revision, we have addressed the remaining comment regarding the biological relevance of ESCRT and COPII downregulation in podocytes. Specifically, we expanded the Discussion to clarify how COPII-mediated ER export, ESCRT-dependent endosomal cargo sorting, lysosome-directed degradation, and autophagy–lysosome function may contribute to proteostasis maintenance and podocyte structural vulnerability.

Please find enclosed the revised manuscript, a marked-up version with tracked changes, and a point-by-point response to the reviewer comments.

Sincerely yours,

Reza Moghadasali

(On behalf of the authors of the manuscript)

Department of Stem Cells and Developmental Biology, Royan Institute for Stem Cell Biology and Technology, Tehran, Iran.

Emails: rezamoghadasali@yahoo.com / s.taleahmad@royan-rc.ac.ir

Reviewer #1 comment:

After the previous round of revisions, the quality of the manuscript has been further improved, and only minor adjustments are needed. The discussion regarding “ESCRT and COPII” could be deepened further. Since GSEA has shown enrichment of these pathways, why is the downregulation of ESCRT and COPII complexes detrimental to podocytes? Further elaboration on this point would be beneficial.

Response:

We thank the reviewer for this constructive suggestion. We have revised and expanded the Discussion to clarify the biological relevance of the ESCRT- and COPII-related GSEA findings.

We now explain that the observed downregulation/negative enrichment of ESCRT- and COPII-associated gene sets may reflect reduced endosomal and secretory trafficking capacity in injured podocytes. This interpretation is biologically relevant because podocytes are highly specialized, polarized epithelial cells whose filtration-barrier function depends on tightly regulated membrane-protein delivery, surface localization, retrieval, recycling, and degradation to maintain slit-diaphragm organization, foot process architecture, and membrane homeostasis. This rationale is supported by prior podocyte studies showing that disruption of endocytic machinery and altered nephrin/podocin turnover impair podocyte foot process integrity and filtration-barrier maintenance. Mechanistically, ESCRT complexes contribute to sorting internalized, often ubiquitinated membrane cargo into multivesicular bodies and lysosomal routes, whereas COPII complexes mediate ER-to-Golgi export of newly synthesized membrane and secretory proteins, supporting secretory delivery and replacement of proteins that require proper processing and surface localization. Therefore, reduced expression of genes associated with these pathways may be consistent with reduced capacity to coordinate both internalized cargo handling and secretory delivery/replacement of membrane-associated proteins, potentially affecting the turnover, localization, and maintenance of slit-diaphragm-associated proteins such as nephrin and podocin during injury. Future studies should directly test this interpretation by assessing ESCRT/COPII component abundance, vesicular trafficking flux, and slit-diaphragm protein localization or turnover in selective podocyte injury models.

The revised text has been added to the Discussion section. [line 339 – line 363]

Reviewer #2:

Comment:

The author has addressed all the issues I raised in my previous round of revisions, and I think this version is ready for acceptance.

Response:

We sincerely thank the reviewer for the careful evaluation of our revised manuscript and for acknowledging that the previous concerns have been addressed. We greatly appreciate the reviewer’s constructive feedback, which helped improve the clarity and quality of the manuscript.

Attachment Submitted filename: Response to Reviewer-second time- PLOS ONE.docx

# Peer review: Decision Letter 2

**DOI:** 10.1371/journal.pone.0352764.r005

**Authors:** Junzheng Yang

16 Jun 2026

Integrated transcriptomic analysis of LMB2-induced podocyte injury identifies conserved inflammatory and adaptive stress responses

PONE-D-26-00699R2

Dear Dr. Moghadasali,

We’re pleased to inform you that your manuscript has been judged scientifically suitable for publication and will be formally accepted for publication once it meets all outstanding technical requirements.

Within one week, you’ll receive an e-mail detailing the required amendments. When these have been addressed, you’ll receive a formal acceptance letter and your manuscript will be scheduled for publication.

An invoice will be generated when your article is formally accepted. Please note, if your institution has a publishing partnership with PLOS and your article meets the relevant criteria, all or part of your publication costs will be covered. Please make sure your user information is up-to-date by logging into Editorial Manager at Editorial Manager® and clicking the ‘Update My Information' link at the top of the page. For questions related to billing, please contact billing support.

If your institution or institutions have a press office, please notify them about your upcoming paper to help maximize its impact. If they’ll be preparing press materials, please inform our press team as soon as possible -- no later than 48 hours after receiving the formal acceptance. Your manuscript will remain under strict press embargo until 2 pm Eastern Time on the date of publication. For more information, please contact onepress@plos.org.

Kind regards,

Junzheng Yang

Academic Editor

PLOS One

Additional Editor Comments (optional):

Reviewers' comments:

Reviewer's Responses to Questions

**Comments to the Author**

1. If the authors have adequately addressed your comments raised in a previous round of review and you feel that this manuscript is now acceptable for publication, you may indicate that here to bypass the “Comments to the Author” section, enter your conflict of interest statement in the “Confidential to Editor” section, and submit your "Accept" recommendation.

Reviewer #1: All comments have been addressed

**********

2. Is the manuscript technically sound, and do the data support the conclusions?

The manuscript must describe a technically sound piece of scientific research with data that supports the conclusions. Experiments must have been conducted rigorously, with appropriate controls, replication, and sample sizes. The conclusions must be drawn appropriately based on the data presented.

Reviewer #1: Yes

**********

3. Has the statistical analysis been performed appropriately and rigorously?

Reviewer #1: Yes

**********

4. Have the authors made all data underlying the findings in their manuscript fully available?

The PLOS Data policy requires authors to make all data underlying the findings described in their manuscript fully available without restriction, with rare exception (please refer to the Data Availability Statement in the manuscript PDF file). The data should be provided as part of the manuscript or its supporting information, or deposited to a public repository. For example, in addition to summary statistics, the data points behind means, medians and variance measures should be available. If there are restrictions on publicly sharing data—e.g. participant privacy or use of data from a third party—those must be specified.

Reviewer #1: Yes

**********

5. Is the manuscript presented in an intelligible fashion and written in standard English?

PLOS ONE does not copyedit accepted manuscripts, so the language in submitted articles must be clear, correct, and unambiguous. Any typographical or grammatical errors should be corrected at revision, so please note any specific errors here.

Reviewer #1: Yes

**********

6. Review Comments to the Author

Please use the space provided to explain your answers to the questions above. You may also include additional comments for the author, including concerns about dual publication, research ethics, or publication ethics. (Please upload your review as an attachment if it exceeds 20,000 characters)

Reviewer #1: All issues raised in the previous rounds of review have been adequately addressed, and the manuscript has been revised accordingly. I therefore recommend acceptance for publication.

**********

7. PLOS authors have the option to publish the peer review history of their article (what does this mean?). If published, this will include your full peer review and any attached files.

If you choose “no”, your identity will remain anonymous but your review may still be made public.

**Do you want your identity to be public for this peer review?**  For information about this choice, including consent withdrawal, please see our Privacy Policy.

Reviewer #1: **Yes:**  Zhui Ke

**********

# Editor report: Acceptance letter

**DOI:** 10.1371/journal.pone.0352764.r006

**Authors:** Junzheng Yang

PONE-D-26-00699R2

PLOS One

Dear Dr. Moghadasali,

I'm pleased to inform you that your manuscript has been deemed suitable for publication in PLOS One. Congratulations! Your manuscript is now being handed over to our production team.

At this stage, our production department will prepare your paper for publication. This includes ensuring the following:

* All references, tables, and figures are properly cited

* All relevant supporting information is included in the manuscript submission,

* There are no issues that prevent the paper from being properly typeset

You will receive further instructions from the production team, including instructions on how to review your proof when it is ready. Please keep in mind that we are working through a large volume of accepted articles, so please give us a few days to review your paper and let you know the next and final steps.

Lastly, if your institution or institutions have a press office, please let them know about your upcoming paper now to help maximize its impact. If they'll be preparing press materials, please inform our press team within the next 48 hours. Your manuscript will remain under strict press embargo until 2 pm Eastern Time on the date of publication. For more information, please contact onepress@plos.org.

You will receive an invoice from PLOS for your publication fee after your manuscript has reached the completed accept phase. If you receive an email requesting payment before acceptance or for any other service, this may be a phishing scheme. Learn how to identify phishing emails and protect your accounts at https://explore.plos.org/phishing.

If we can help with anything else, please email us at customercare@plos.org.

Thank you for submitting your work to PLOS ONE and supporting open access.

Kind regards,

PLOS ONE Editorial Office Staff

on behalf of

Director Junzheng Yang

Academic Editor

PLOS One
